# Supplementary figures and images for: A registered report of a crossover study on the effects of face masks on walking adaptability in people with Parkinson’s disease and multiple sclerosis
Source: PLoS One. 2023 Jun 29;18(6):e0286402. doi: 10.1371/journal.pone.0286402 (PMC10309975; doi:10.1371/journal.pone.0286402)

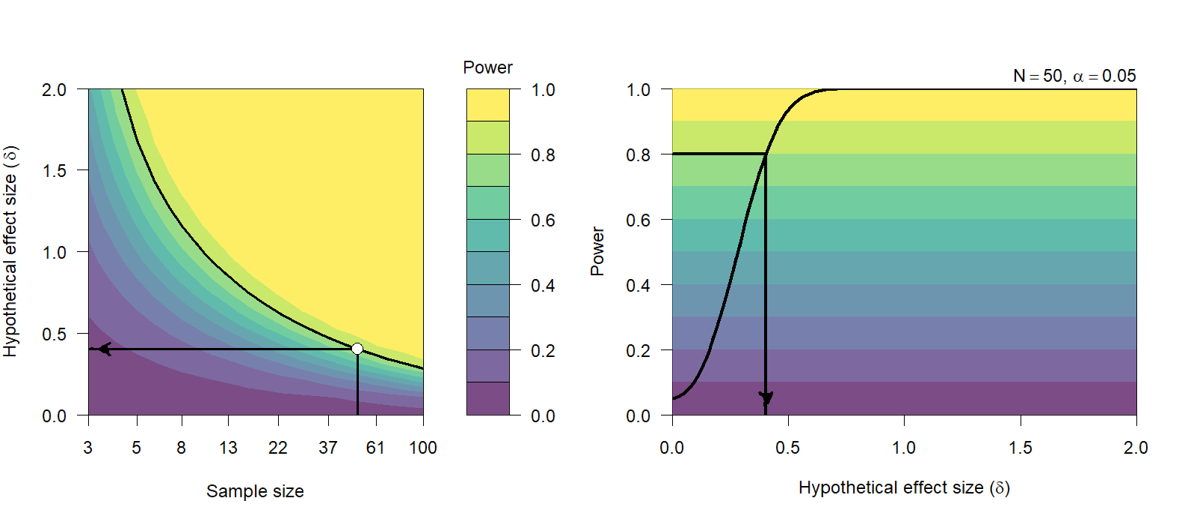

Supplement: S1 Fig — (TIF) [file pone.0286402.s003.tif]
