# Supplementary material for: A registered report of a crossover study on the effects of face masks on walking adaptability in people with Parkinson’s disease and multiple sclerosis
Source: PLoS One. 2023 Jun 29;18(6):e0286402. doi: 10.1371/journal.pone.0286402 (PMC10309975; doi:10.1371/journal.pone.0286402)
Supplement: S1 File — (DOCX) [file pone.0286402.s004.docx]

**Einfluss des Tragens eines Mund-Nasen-Schutzes auf die Ganganpassungsfähigkeit von Menschen mit einer neurologischen Erkrankung**

**Version 1, Juni 2021**

**Hauptansprechpartnerin:**

Mareike Eschweiler, MSc.

Leitung Therapiewissenschaften

Neurologisches Rehabilitationszentrum „Godeshöhe“ e. V. (NRZ „Godeshöhe“)

Waldstraße 2-10

53177 Bonn

Telefon +49 228 381-559

Fax +49 228 381-318

E-Mail m.eschweiler@godeshoehe.de

**Antrag auf Erteilung eines Votums der Ethikkommission der Medizinischen Fakultät der Universität Bonn**

Zur Durchführung eines medizinischen-wissenschaftlichen Vorhabens, welches nicht die klinische Prüfung eines Arzneimittels oder eines Medizinproduktes beinhaltet.

# A. FORMALES

## Bezeichnung des Vorhabens

Einfluss des Tragens eines Mund-Nasen-Schutzes auf die Ganganpassungsfähigkeit von Menschen mit einer neurologischen Erkrankung

## Antragsteller*innen, verantwortliche Leiter*innen und Kooperationspartner*innen:

### Antragsteller*in und verantwortliche*r Projektleiter*in:

Mareike Eschweiler, M.Sc.

Leitung Therapiewissenschaften

NRZ „Godeshöhe“ e.V.

Waldstraße 2-10

53177 Bonn

Telefon +49 228 381 – 559

E-Mail [m.eschweiler@godeshoehe.de](mailto:m.eschweiler@godeshoehe.de)

### Verantwortliche*r medizinische*r Leiter*in:

Prof. Dr. med. Hans Karbe

Ärztlicher Direktor

NRZ „Godeshöhe“ e.V.

Akademischer Lehrbeauftragter für Rehabilitationsmedizin

Medizinische Fakultät

Rheinische Friedrich-Wilhelms-Universität Bonn

Waldstraße 2-10

53177 Bonn

Telefon +49 228 381 - 206 (Sekretariat: +49 228 381 - 207)

E-Mail h.karbe@godeshoehe.de; (Sekretariat: [karbe.office@godeshoehe.de](mailto:karbe.office@godeshoehe.de))

Hiermit erkläre ich mein Einverständnis zur Durchführung der Studie: „Einfluss des Tragens eines Mund-Nasen-Schutzes auf die Ganganpassungsfähigkeit von Menschen mit einer neurologischen Erkrankung“ im NRZ „Godeshöhe“, Bonn-Godesberg.

Prof. Dr. med. Hans Karbe

Ärztlicher Direktor des NRZ „Godeshöhe“

### Kooperationspartner*in

Dr. Eleftheria Giannouli

Universität Basel

Departement für Sport, Bewegung und Gesundheit

Abteilung für Sport- und Bewegungsmedizin

Birsstrasse 320 B

4052 Basel, Schweiz

Telefon +41 61 207 47 45

E-Mail eleftheria.giannouli@unibas.ch

Web <https://dsbg.unibas.ch>

Chris McCrum, PhD

Assistant Professor of Human Movement Sciences

Universität Maastricht

Abteilung für Ernährungs- und Bwegungswissenschaften

Universiteitssingel 50

6229 ER Maastricht, Niederlande

E-Mail chris.mccrum@maastrichtuniversity.nl

Web <https://www.maastrichtuniversity.nl/chris.mccrum>

Die Kooperationen mit Dr. Giannouli und Dr. McCrum bestehen darin, dass die geplante Studie gemeinsam entwickelt wurde. Im weiteren Verlauf des Projektes ist ein Austausch über die Daten, die Analyse und die Analyseergebnisse sowie die gemeinsame Erstellung von Manuskripten zu Publikationszwecken geplant.

Dr. McCrum hat bereits einen Beitrag im BMJ (McCrum, 2020) zum wissenschaftlichen Diskurs über dieses Thema beigetragen und sieht in der klinischen Überprüfung eine relevante Ergänzung der bisher theoretisch geführten Diskussion.

Es besteht bereits ein enger fachlicher Austausch mit Dr. Giannouli für anderweitige Studienprojekte.

### Weitere beteiligte Personen

Dipl. Psych. Jochen Saliger

Leitung kognitive Rehabilitation

NRZ „Godeshöhe“ e.V.

Waldstraße 2-10

53177 Bonn

Telefon +49 228 381-702

E-Mail [j.saliger@godeshoehe.de](mailto:j.saliger@godeshoehe.de)

Peter Wendland

Leitung Physiotherapie

NRZ „Godeshöhe“ e.V.

Waldstraße 2-10

53177 Bonn

Telefon +49 228 381-989

E-Mail [p.wendland@godeshoehe.de](mailto:p.wendland@godeshoehe.de)

Florian Wolf, M.A.

Wissenschaftlicher Mitarbeiter

Sporttherapeut

NRZ „Godeshöhe“ e.V.

Waldstraße 2-10

53177 Bonn

Telefon +49 228 381 - 481

E-Mail [f.wolf@godeshoehe.de](mailto:f.wolf@godeshoehe.de)

## Art und Zahl der Prüfstellen

Das geplante Studienvorhaben wird als monozentrische Studie durchgeführt.

Es besteht lediglich eine fachliche Kooperation, wie oben beschrieben (2.3.) mit Dr. Giannouli und PD Dr. McCrum.

## Kostenträger

Die Studie wird aus Eigenmitteln des NRZ „Godeshöhe“ finanziert.

Zusätzlich wurde am 30.05.2021 ein Drittmittelantrag beim Förderverein NRZ Bonn e.V., c/o NRZ Godeshöhe e.V., Waldstraße 2-10, 53177 Bonn gestellt (Entscheidung noch ausstehend).

## Entscheidung der Ethikkommissionen in derselben Sache

Es wurde kein Antrag bei einer anderen Ethik-Kommission gestellt.

# B UNTERSUCHUNGSBESCHREIBUNG

## Wissenschaftliche Beschreibung des Vorhabens

### 1.1 Stand der Forschung und wissenschaftlicher Hintergrund

Die Covid-19 Pandemie stellt die Welt seit Anfang 2020 immer wieder vor neue Herausforderungen (WHO, 2021; Bundesregierung, 2020a; 2021). Insbesondere die Eindämmung der Ansteckungsraten und die Abwendung einer Überlastung des Gesundheitssystems bildet dabei den Fokus aller Bemühungen (Bundesregierung, 2020a; 2020c). Die dazu getroffenen und immer wieder angepassten Maßnahmen beeinflussen das tägliche Leben vieler Menschen (Bundesregierung, 2020a; 2020b; 2020c; 2020d; 2020e, 2020f; 2020g; 2020h; 2021d). Trotz optimistisch stimmendem Impffortschritt, ist das Ende der Pandemie sowie der Maßnahmen zur Virusprävention derzeit nicht absehbar (UN, 2021; Freund, 2021; Callisaya et al., 2020). Immer wieder kommt es zu neuen Infektionswellen (Bundesregierung, 2020h; 2021a; 2021d RKI, 2021) und damit verbunden zu verschärften Präventionsmaßnahmen (Bundesregierung, 2020i; 2021d).

Insbesondere die „Abstand-Hygiene-Alltagsmasken“-Regeln (Bundesregierung, 2020d; 2020e; 2020f; WHO, 2020) sowie das regelmäßige Durchlüften von geschlossenen Räumen (Bundesregierung, 2020g) haben sich als effektiver, individueller Beitrag zur Eindämmung des Infektionsgeschehens bewährt. Die Empfehlung bzw. Verordnung zum Tragen von Alltagsmasken wurde inzwischen vielerorts durch die Pflicht zum Tragen einer chirurgischen oder FFP2-Masken ersetzt (Bundesregierung, 2020d; 2020j; 2021a; 2021b; 2021c).

Jegliche Masken, unabhängig ob medizinisch oder nicht-medizinisch, bedecken einen wesentlichen Teil des Gesichts. Durch ihre Form ragen sie durchaus in das untere Gesichtsfeld hinein (Kal et al., 2020a, Klatt et al., 2021). Es ist denkbar, dass diese Gesichtsfeldbeeinträchtigung einen Einfluss auf die Perzeption visueller Reize hat (Buckley et al., 2011; Rietdyk & Rhea, 2011), denn dieser Bereich des unteren Gesichtsfeldes liefert wichtige Informationen für eventuell nötige Ganganpassungen (z.B. das Ausweichen oder Übersteigen von Hindernissen) und sicheres Gehen (Buckley et al., 2011). Eine Einschränkung in diesem Bereich durch das Tragen eines MNS könnte zu einer Beeinträchtigung der Gangleistung und –sicherheit und möglicherweise zu einem gesteigerten Stolper- und Sturzrisiko beitragen könnte (Klatt et al., 2021; Callisaya et al., 2020; Rietdyk & Rhea, 2011; Lord et al., 2002). Kal und Kollegen vermuten, dass dies einen Einfluss auf die Gangsicherheit älterer Menschen haben könnte (Kal et al, 2020a). Diese Vermutung wird von der Studie von Lord und Kolleg*innen (2002) insofern gestützt, als dass sie zeigen konnten, dass bereits das Tragen multifokaler Brillen die Gangsicherheit reduzierte. Ältere Menschen, ab einem Alter von > 65 Jahren, haben ein gesteigertes Sturzrisiko (Rapp et al., 2014), so dass es sehr wahrscheinlich ist, dass durch weitere Einschränkungen, wie das Tragen eines MNS, das Risiko zusätzlich erhöht wird. Noch gravierender sind diese Negativfolgen möglicherweise für die Population neurologisch vorerkrankter Menschen (Klatt et al., 2021; Callisaya et al., 2020), die auch ohne MNS bereits ein vielfach erhöhtes Sturzrisiko im Vergleich zur Durchschnittsbevölkerung haben (Beghi et al., 2018).

Bei Menschen mit einer neurologischen Grunderkrankung sind u.a. Gleichgewichts-, Geh- und Reaktionsfähigkeit bereits durch die neurologischen Störungen beeinträchtigt, so dass die zusätzlichen Einschränkungen des Gesichtsfeldes und die damit verbundene reduzierte sensorische Information durch das Tragen eines MNS möglicherweise nicht mehr so leicht kompensiert werden können und somit das Stolper- und Sturzrisiko sich massiv erhöhen könnte (Klatt et al., 2021; Kal et al., 2020a; Yakubovich et al., 2020; Callisaya et al., 2020).

Eine erste wissenschaftliche Debatte ist darüber entstanden, was betroffenen Menschen geraten werden könnte, um ein vermeintlich erhöhtes Sturzrisiko zu kompensieren. Kal et al. (2020a; 2020b) schlagen dazu ein reduziertes Gehtempo vor. Sie weisen darauf hin, dass der intuitive und teilweise angeratene Blick nach unten möglicherweise die Gangsicherheit zusätzlich negativ beeinflusst (Kal et al., 2020a). Callisaya et al., 2020 argumentieren, dass ein verlangsamtes Gehtempo ebenfalls die Gangsicherheit reduzieren könnte. McCrum (2020) weist darauf hin, dass verschiedene Aspekte der posturalen Kontrolle und mechanischen Stabilität von Bedeutung sind und argumentiert, dass die Reduzierung des Gehtempos durchaus angemessen sein könnte. Er ergänzt das dies möglicherweise sogar dem Sturz nach vorne vorbeugen könnte (McCrum, 2020). Insgesamt wird aus der Diskussion deutlich, dass eindeutige und personalisierte Empfehlungen derzeit schwierig sind. Hierfür fehlt auch die tatsächliche praktische Untersuchung, die Prävalenzen und ggf. erste Einschränkungen aufzeigen würde und von der sich erste Empfehlungen ableiten ließen. Daher soll das geplante Studienvorhaben diese Aspekte in einer neurologischen Kohorte untersuchen. Callisaya et al. (2020) weisen in ihrem Beitrag außerdem darauf hin, dass die Aufklärung von möglicherweise betroffenen Populationen ein essentieller Schritt ist. Die Arbeitsgruppe führt aus, dass das Wissen um mögliche Risikosituationen ein wichtiger Präventionsaspekt ist. Für derartig edukative Ansätze ist es jedoch relevant zu wissen, wer zu den Betroffenen tatsächlich zählt. Hierfür ist es relevant, diese Frage nicht nur theoretisch zu erörtern, sondern tatsächliche Prävalenzen beispielsweise in der neurologischen Population zu erheben. Denn diese, eher theoriegeleitetet Wissenschaftsdiskussion lässt die Frage offen, wie prävalent das vermutetet Phänomen in einer Kohorte neurologisch erkrankter Menschen tatsächlich ist. Daher soll das geplante Studienvorhaben untersuchen, ob das „Tragen eines MNS“ gegenüber dem „nicht Tragen eines MNS“ einen Einfluss auf den Gang und die Gangsicherheit hat.

### 1.2. Studienziel:

Um dies zu überprüfen soll ein standardisierter Test zur Messung der Anpassungsfähigkeit des Ganges (CGait) auf einem VR-basierten Laufband (C-Mill+VR by Motek) einmal mit und einmal ohne Maske sowie spezifische klinische Tests (10 Meter Gehtest, 10mWT; Timed Up and Go Test, TUG, TUG mit Doppelaufgabe (dual task, TUG+DT) und das Treppen auf- und absteigen) bei 50 Menschen mit einer neurologischen Erkrankung durchgeführt werden. Außerdem sollen sich die Teilnehmer*innen, anhand standardisierter Fragen, subjektiv zu ihren Erfahrungen beim Gehen mit und ohne Maske äußern.

Diese Studie kann den bestehenden theoretischen wissenschaftlichen Diskurs um klinische Daten für Menschen mit einer neurologischen Erkrankung ergänzen und so einen wichtigen Beitrag zu einer bestehenden Wissenschaftsdebatte leisten. Darüber hinaus würden diese Erkenntnisse den betroffenen Menschen insofern dienen, als dass es eine wissenschaftlich begründete Anerkennung ihrer zusätzlichen Einschränkungen möglich wäre. Auch könnte dies, in Anlehnung an die Forderungen von Callisaya und Kolleg*innen (2020) sowie von Kal und Kolleg*innen (2020b), die Basis für weitere klinische Untersuchungen hinsichtlich möglicher Kompensationen oder Empfehlungen für Betroffen darstellen.

### 1.3. Fragestellungen und Hypothesen:

Die wesentliche Fragestellung, die in der geplanten Studie geklärt werden soll, ist ob das Tragen des MNS (FFP-2 Maske) einen Einfluss auf die Anpassungsfähigkeit des Ganges (gemessen mit dem Performanz-Wert (%) je CGait Subtest für jedes der beiden Schwierigkeitsniveaus) bei Menschen mit einer neurologischen Erkrankung hat (*Fragestellung 1*). Es wird angenommen, dass Menschen mit einer neurologischen Erkrankung (Multiple Sklerose oder Parkinson) in einzelnen oder allen CGait Subtests signifikant schlechter abschneiden, wenn sie einen MNS tragen, als wenn sie keinen tragen (*Hypothese 1*).

Da es jedoch bei klinisch beobachteter Verbesserung nicht nur um standardisierte, objektive Messungen gehen kann, sondern der Selbsteinschätzung von Patient*innen ebenfalls ein hoher Stellenwert einzuräumen ist, soll außerdem geklärt werden, ob das Tragen eines MNS einen Einfluss auf die subjektiv empfundene Gehfähigkeit der Patient*innen hat (*Fragestellung 2*), da angenommen werden kann, dass Menschen mit einer neurologischen Erkrankung eine Beeinträchtigung ihrer Gehfähigkeit durch das Tragen eines MNS bemerken (*Hypothese 2*). Darüber hinaus ist bekannt, dass die subjektive Wahrnehmung der Patient*innen einen Einfluss auf die Gangleistung haben könnte (Hoogkamer et al., 2015; Rosengren et al., 1998), deshalb ist es relevant, diese zu erfragen.

Neben diesen beiden Hauptaspekten der Studien, soll darüber hinaus explorativ untersucht werden, ob sich in diesen klinischen Tests sowie dem Treppe auf- und abgehen ebenfalls ein Einfluss durch das Tragen eines MNS zeigt (*Fragestellung 3*). Möglicherweise zeigen Menschen mit einer neurologischen Erkrankung auch in gängigen klinischen Tests zur Messung der Gehfähigkeit (Treppentest, TUG, TUG+DT, 10mWT) Auffälligkeiten (*Hypothese 3*). Diese, wenn auch explorative Erkenntnis, würde für alle Kliniker*innen, die kein CMill+VR zur Verfügung haben relevante Informationen für die klinische Routinetestung liefern.

## Prüfplan

### 2.1. Studienablauf

Im Rahmen des Projektes soll untersucht werden, inwiefern das Tragen eines MNS bei Menschen mit einer neurologischen Erkrankung die Ganganpassungsfähigkeit (CGait) und die Mobilität (klinische Parameter) beeinflusst.

Dazu werden 50 gehfähige Patient*innen die ihren Rehabilitationsaufenthalt im neurologischen Rehabilitationszentrum „Godeshöhe“ (NRZ) absolvieren werden angesprochen, ob sie bei dieser Studie mit einem Messzeitpunkt teilnehmen wollen. Sie werden mündlich und schriftlich über das Studienvorhaben aufgeklärt. Sollten sie teilnehmen wollen, müssen sie schriftlich ihr Einverständnis zur Teilnahme und zur Datenverarbeitung erteilen.

Danach werden einige soziodemographische (Alter, Geschlecht, Ausbildungsjahre, Körpergröße, Schuhgröße, Gewicht, BMI, Brillenträger*in, Art der Visuseinschränkung), klinische (Diagnose, Jahr der Diagnosestellung, Erkrankungsschwere, Sturzereignisse in den letzten 12 Monaten, subjektive Sturzangst, Medikamente ggf. Schrittmachereinstellungen bei Menschen mit Parkinson) erfasst. Diese werden erhoben, um die Stichprobe dezidiert beschreiben zu können.

Als primärer Endpunkt wird der CGait Gehtest einmal mit und einmal ohne Maske von jede*r Teilnehmer*in absolviert. Die Reihenfolge „Maske/ keine Maske“ wird pro Teilnehmer*in randomisiert. Unter allen Teilnehmer*innen wird es die Reihenfolge „Maske/ keine Maske“ gleich häufig geben wie die Reihenfolge „keine Maske/ Maske“. Der CGait Test ist ein Standardtest, der ins Diagnostikprogramm des C-Mill by Motek VR Laufbandes (DIH GmbH, Köln, GER) integriert ist. Er dauert 20 Minuten und umfasst eine Reihe von Aufgaben zur Überprüfung der Anpassungsfähigkeit des Ganges, u.a. Übersteigen von Hindernissen (s. Abschnitt 2.2.1). Zusätzlich werden Standardtests der motorischen Diagnostik (10mWT, TUG, TUG+DT und Treppentest) erhoben. Abbildung 1 veranschaulicht den geplanten Messablauf. Kapitel 2.2. gibt eine Übersicht über die geplante Datenerhebung


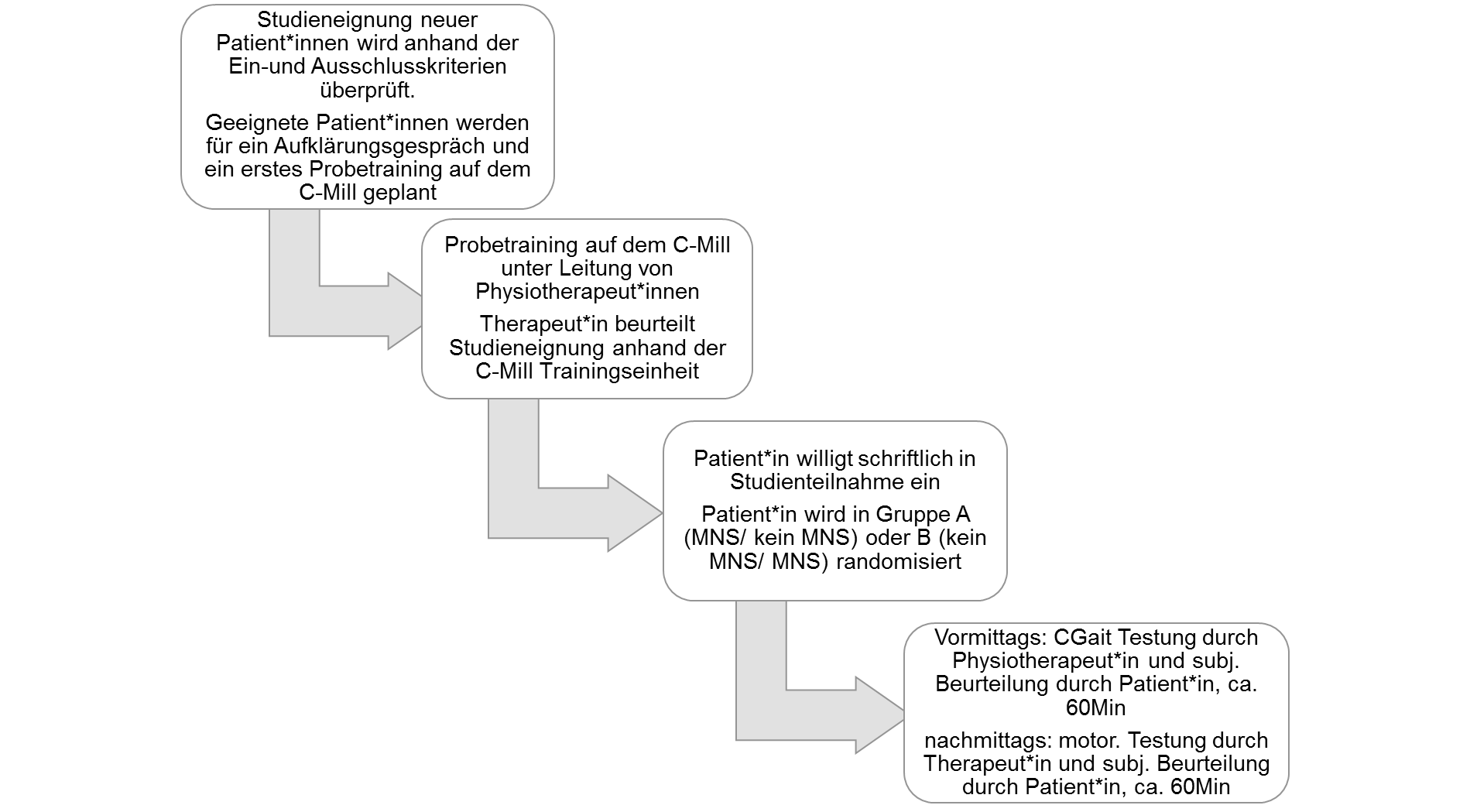


Abbildung 1: Schematische Darstellung des Studienablaufes

### 2.2. Assessments

Die zu erhebenden Daten werden durch Befragung (soziodemografische und klinische Daten sowie subjektive Einschätzungen) und einfache klinische Untersuchung (motorische Gang- und Gleichgewichtstests) gewonnen. Tabelle 1 zeigt zusammenfassend die für die Studie geplanten Assessments. Die meisten Daten, die ihm Rahmen des Projekts gesammelt werden sollen werden in der gängigen Routine erhoben. Die zusätzliche Belastung für die Proband*innen ist folglich gering (85 Min.).

Die soziodemografischen und klinischen Daten können zum größten Teil aus der digitalen Akte entnommen werden. Sie werden jedoch im persönlichen Gespräch mit den Patient*innen sowie den behandelnden Ärzt*innen verifiziert (Befragung).

Die Erfassung der zurückliegenden Sturzereignisse und sturzassoziierten Selbstwirksamkeit erfolgt mittels standardisierter Fragebögen. Die Selbsteinschätzungen der Proband*innen erfolgt mit selbst entwickelten Befragungen.

Bei der motorischen Testung werden mittels standardisierter Assessments für die neurologische Rehabilitation verschiedene Facetten der Mobilität erfasst. Die Testung wird von erfahrenen Physiotherapeut*innen und/ oder Sportwissenschaftler*innen durchgeführt, die mit den Testungen seit vielen Jahren vertraut sind. Die Assessments können aufgrund von Personalressourcen nicht verblindet sein.

Tabelle 1: Geplante Assessments

| **Tests** | **Erhebungsform** | **Dauer** | **Routine** | **T0** |
| --- | --- | --- | --- | --- |
| Soziodemografische Daten (Gesamtdauer ca. 7 Min.) | | | | |
| Alter in Jahren | Akte/ Befragung | < 1Min. | x | x |
| Geschlecht | Akte/ Befragung | < 1Min. | x | x |
| Ausbildungsjahre | Akte/ Befragung | < 1Min. | x | x |
| Gewicht in Kg | Akte/ Befragung | < 1Min. | x | x |
| Körpergröße in cm | Akte/ Befragung | < 1Min. | x | x |
| Body Maß Index, BMI | Wird aus Körpergröße und Gewicht errechnet (Formel der WHO^[[1]](#footnote-1)^) |  |  |  |
| Schuhgröße | Befragung | < 1Min. |  | x |
| Brillenträger*in | Befragung | < 1Min. |  | x |
| Art der Visuseinschränkung | Befragung | < 1Min. |  | x |
| Klinische Daten (Gesamtdauer ca. 13 Min.) | | | | |
| Diagnose inkl. Verlaufsform/ Typ | Akte/ Befragung | < 1Min. | x | x |
| Zeit seit Diagnosestellung (in Monaten) | Akte/ Befragung | < 1Min. | x | x |
| Schweregrad der Erkrankung (Expanded Disability Status Scale, EDSS Kurtzke, *Neurol*, 1983; Hoehn & Yahr Skala  Hoehn & Yahr, Neurol, 1967) | Akte/ Erhebung bei motor. Testung | 5 Min. | x | x |
| Sturzereignisse in den letzten 12 Monaten | Befragung | < 1Min. |  | x |
| Sturzassoziierte Selbstwirksamkeit (Activities-Specific Balance Confidence Scale, ABC *Schott, Z Gerontol Geriat, 2008*) | Befragungen | 5 Min | x | x |
| Motorische Daten (Gesamtdauer 45 Min., je 2x) | | | | |
| Ganganpassungsfähigkeit (CGait, Timmermanns et al., *Phys Ther*, 2019) | Motor. Test | 20 Min. | x | x |
| 10m Gehstrecke (10mWT; ) | Motor. Test | 5 Min. | x | x |
| Funktionelle Mobilität und Gleichgewicht (Timed Up and Go Test, TUG Mathias et al., *Arch Phys Med Rehabil*, 1986) | Motor. Test | 5 Min. |  | x |
| Gehen mit Doppelaufgabe (TUG mit Doppelaufgabe, TUG+DT Hofheinz et al., *HeilberufeScience*, 2011) | Motor. Test | 10 Min. |  | x |
| Treppe auf- und absteigen (acending/ decending stair test Nightingale, *J Rehabil Res Dev*, 2014) | Motor. Test | 5 Min. |  | x |
| Proband*innen-Befragung (Gesamtdauer 20 Min.) | | | | |
| Subjektive Einschätzung von Proband*innen zur Performanz mit/ ohne Maske | Befragung | 15 Min. |  | x |
| Subjektive Einschätzung von Proband*innen zum C-Mill | Befragung | 5 Min. |  | x |

*Gesamtdauer*: 150 Min. davon ausschließlich studienbedingte Testung ca. 85 Min.

*Für die Assessments gilt*: Sofern sich individuelle Einschränkungen aufgrund des Krankheitsbildes ergeben oder die Patient*innen aus irgendwelchen Gründen, die sie nicht näher benennen brauchen, eine Pause benötigen oder eine Fortsetzung der Testung/ der Befragung nicht wünschen, wird die Testung/ Befragung unter- bzw. abgebrochen.

#### 2.2.1. Primärer Endpunkt

Der zentrale Parameter der Studie ist die Leistung (Performanz-Wert in %) pro CGait Subtest in den beiden Schwierigkeitsstufen des CGait (sofern für die Patient*innen möglich).

Der CGait Test misst die Anpassungsfähigkeit des Ganges mittels sechs verschiedenen Subtests auf dem CMill + VR Laufband. Eine umfassende Beschreibung des CGait liefert die Publikation von Timmermans et al. (2019).

Während des Tests sind die Proband*innen über einen Gurt gesichert und die komfortable Gehgeschwindigkeit wird zum Beginn des Tests ermittelt. Diese Geschwindigkeit wird über alle Aufgaben hinweg beibehalten. Im geplanten Studienvorhaben wird sie für den Test mit und ohne Maske ebenfalls beibehalten. Alle Aufgaben des CGait werden von den Proband*innen zunächst mit einem leichten und dann mit einem schweren Schwierigkeitsgrad absolviert. Die Instruktion der Proband*innen erfolgt sowohl über einen integrierten Bildschirm als auch durch die Therapeut*innen.

Die Performanz für die sechs Testaufgaben (Abb. 2) „goal-directed stepping“, „tandem walking“, „obstacle avoidance“, „slalom walking“, „speed adaptations“, wird gemessen. Sie ermittelt sich über den Anteil der Schritt bei denen der Druckmittelpunkt während der mittleren Standbeinphase (± die Hälfte der Fußgröße bei „goal-directed stepping“ und „obstacle avoidance“) innerhalb der projizierten Fläche lag. Bei der Aufgabe „walking with suddenly shifting obstacles and targets” wird der gewichtete Durchschnitt der korrekten Schritte und der korrekt ausgewichenen Hindernisse berechnet. Es ergibt sich für jede der sechs Aufgabe ein Performanz-Wert zwischen 0 und 100%.

Abbildung 2: Sechs Untertests des CGait Test aus Timmermans et al., 2019

Die Performanz-Werte der einzelnen Subtests werden pro Bedingung (1: mit MNS, 2: ohne MNS) und für jede Schwierigkeitsstufe (leicht, schwer) verglichen.

#### 2.2.2. Sekundäre Endpunkte

Da es jedoch bei klinisch beobachteter Verbesserung nicht nur um standardisierte, objektive Messungen gehen kann, sondern der Selbsteinschätzung von Patient*innen ebenfalls ein hoher Stellenwert einzuräumen ist und diese möglicherweise ebenfalls einen Einfluss auf die Leistung hat, wird die subjektive Einschätzung von Patient*innen, ob das Tragen eines MNS einen Einfluss auf die subjektiv empfundene Gehfähigkeit hat, mittels Befragung erfasst und deskriptiv ausgewertet.

Als weitere sekundäre Endpunkte werden die zentralen Tendenzen der Durchführungszeiten pro Bedingung („Tragen eines MNS“ und „nicht Tragen eines MNS“) für die jeweiligen klinischen Parameter (10mWT, TUG, TUG+DT und Treppentest) verglichen.

### 2.3. Verblindung

Es findet keine Verblindung von Test- und/oder Trainingsleiter*innen statt.

### 2.4. Randomisierung

Die Proband*innen werden zufällig den Bedingungen, also der Reihenfolge der Testungen (Bedingung „mit Maske/ ohne Maske“ oder „ohne Maske/ mit Maske“) zugeordnet. Beide Bedingungen sollen unter allen Teilnehmer*innen gleich oft vorkommen.

### 2.5. Statistische Auswertung

Die erhobenen, pseudonymisierten Daten werden digital erfasst (Vier-Augen-Prinzip).

Die Datenanalyse wird mit gängiger Analysesoftware IBM SPSS Statistics 27.0 (Armonk, NY) und der open source Software R (R Foundation for Statistical Computing, Wien) durchgeführt.

Zu Beginn der Analyse werden die Daten bezüglich der Erfüllung geforderter Voraussetzungen überprüft. Für die Überprüfung werden die jeweils typischen statistischen Verfahren eingesetzt.

Zur Beschreibung der Stichprobe werden Häufigkeiten, Mediane (mit Minimum und Maximum) oder Mittelwerte (mit Standardabweichung) berichtet, je nach Angemessenheit.

Zur Beantwortung der *Fragestellung 1*, ob das Tragen des MNS (FFP-2 Maske) einen Einfluss auf die Anpassungsfähigkeit des Ganges (gemessen mit den Performanz-Werten der CGait Subtests in beiden Schwierigkeitsstufen) bei Menschen mit einer neurologischen Erkrankung hat, wird, je nach Angemessenheit ein parametrischer oder nicht-parametrischer Innergruppenvergleich gerechnet.

Die *2. Studienfrage*, ob das Tragen eines MNS einen Einfluss auf die subjektiv empfundene Gehfähigkeit der Patient*innen hat, wird mittels deskriptiver Statistik (Häufigkeitstabellen) dargestellt.

Neben diesen beiden zentralen Studienfragen, soll die *Fragestellung 3* der Studie, ob sich in den klinischen Tests zur Messung der Gehfähigkeit (10mWT, TUG, TUG+DT und Treppentest) ebenfalls ein Einfluss durch das Tragen eines MNS zeigt, explorativ untersucht werden. Dazu werden, je nach Angemessenheit parametrische oder nicht-parametrische Innergruppenvergleiche für jeden Untertest gerechnet.

Für alle Innergruppenvergleiche wird das Signifikanzniveau wird bei *p* ≤ 0,05 festgelegt.

### 2.6. Veröffentlichungen und Registrierung der Studie

Die Studie wird als Registered Report bei einer an dem Center for Open Science teilnehmenden Fachzeitschrift eingereicht (z.B. BMC Medicine). Eine Registered Report-Einreichung bedeutet, dass das Protokoll (Einleitung und Methode) im „Stage 1“ durch den Peer Review Prozess geht, bevor Daten gesammelt werden. Nach einer „In Principle Acceptance“ dürfen die Forscher dann mit der Studie anfangen. In “Stage 2” sind die Ergebnisse und Diskussion peer reviewed und solange die Forscher die vorher genehmigte Methode und Protokolle durchgeführt haben, wird die Studie in der Zeitschrift veröffentlicht. Mehr zu Registered Reports findet mann hier: <https://www.cos.io/initiatives/registered-reports>

Die Ergebnisse des Projektes sollen auch auf Kongressen (WFNR World Congress oder Deutschen Gesellschaft für Neurorehabilitation e. V.) sowie im Rahmen interner Schulungen vorgestellt werden.

Die Studie wird im Deutschen Register Klinischer Studien (DRKS) registriert. Die Finalisierung der Registrierung erfolgt nach positiven Votum durch die Ethikkommission (beim DRKS nicht mehr anders möglich).

## Vorgesehene Gesamtdauer

Das nachfolgende Schema (Abb. 1) stellt den geplanten Prüfplan des Studienvorhabens dar.

Abbildung 3: Prüfplan zum Studienvorhaben

## Probandenauswahl

### 4.1. Probandenrekrutierung

Die Rekrutierung von neurologischen Patient*innen, erfolgt während ihres stationären Rehabilitationsaufenthaltes im NRZ „Godeshöhe“.

### 4.2. Ein- und Ausschlusskriterien

Die Patient*innen werden nach folgenden Kriterien in die Studie eingeschlossen oder müssen von ihr ausgeschlossen werden:

| Einschlusskriterien | Ausschlusskriterien |
| --- | --- |
| - eine durch die*en behandelnde*n Ärztin/ Arzt gesicherte neurologische Diagnose (Parkinson oder Multiple Sklerose) - die Proband*innen sind aufklärungsfähig - die Proband*innen geben ihr schriftliches Einverständnis zur Studienteilnahme | - keine bestehenden Kontraindikationen gegen die Testung auf dem C-Mill+VR Laufband - sonstigen neurologischen oder psychiatrischen Erkrankungen, die die Aufklärungsfähigkeit, das Testverständnis sowie die Trainingssicherheit beeinflussen oder einen zusätzlichen Einfluss auf die Motorik haben - unzureichende Deutschkenntnisse, um Anweisungen und Testungen zu folgen - visuelle oder auditive Einschränkung (kann Aufgaben nicht folgen) - >135kg Körpergewicht und >2m Körpergröße (Gerätebeschränkung) - Offene Wunden im Bereich des C-Mill Sicherungsgurtes |

### 4.3. Stichprobenumfang

Das geplante Vorhaben soll mit der Unterstützung von 50 Proband*innen umgesetzt werden. Dies wird als eine machbare Stichprobengröße für den beabsichtigen Studienzeitraum angesehen. Hierzu wurde keine a priori Fallzahlkalkulation durchgeführt, da bisher hierzu keine Studien vorliegen, auf deren Basis notwendige à priori Annahmen getroffen werden können. Klinisch relevante Mindestunterschiede für die angestrebten Ergebnisparameter sind, aufgrund der Neuartigkeit der Thematik, noch bisher nicht etabliert. Auch hier wird die geplante Studie einen relevanten Beitrag leisten.

Nichtsdestotrotz, zeigte eine Sensitivitäts-Power-Analyse, dass mit dieser machbaren Stichprobengröße, die Analyse eine Power von 0,8 haben wird, eine Effektstärke von *d* = 0,4 und eine Power von 0,95 haben wird, Effektstärken von *d* = 0,52 zu identifizieren (s. Abb.)


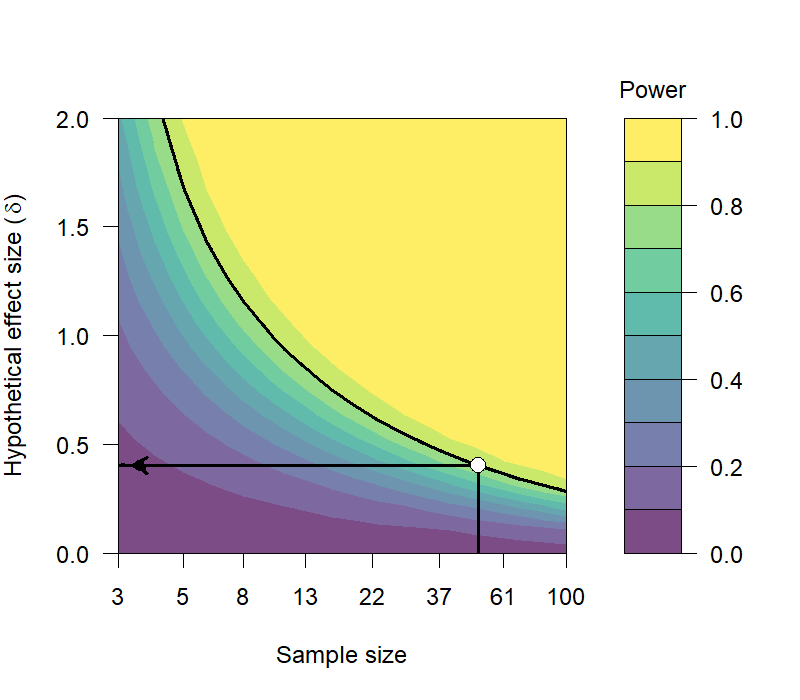

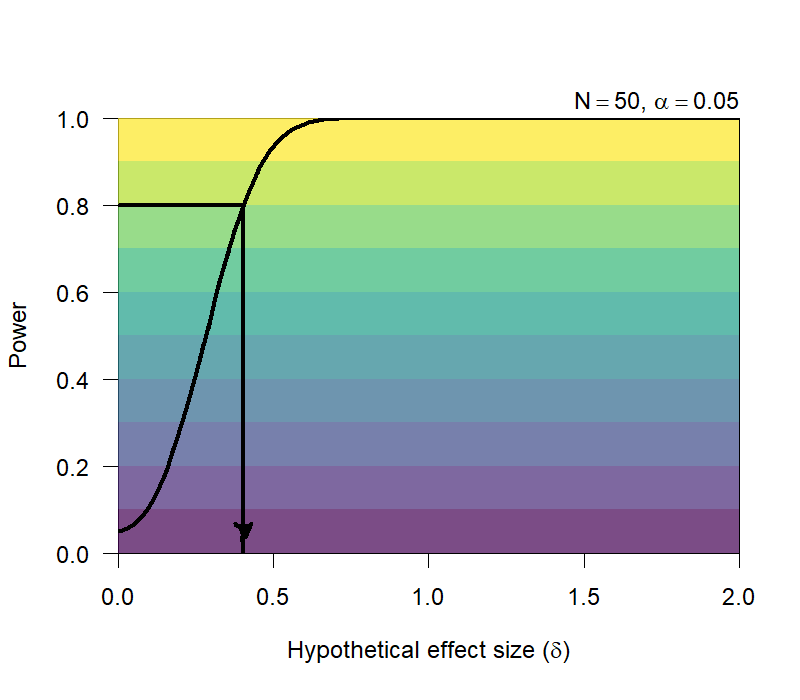
Abbildung 4: Ergebnisse der Sensitivitäts-Power-Analyse.

### 4.4. Aufklärung und Studieneinverständnis

Geeignete Patient*innen werden kontaktiert, aufgeklärt und das schriftliche Einverständnis wird eingeholt. Nachfolgend wird das entsprechende Vorgehen detailliert beschrieben.

#### 4.4.1. Kontaktaufnahme

Bei Zugang/ Aufnahme von neurologischen Patient*innen, wird die mögliche Eignung nach Ein- und Ausschlusskriterien anhand der Akte festgestellt.

Potentiell geeignete Proband*innen werden im Rahmen eines persönlichen Gespräches zwischen Proband*in und Studienleitung über das Studienvorhaben informiert und erhalten bei Interesse die schriftliche Information für Proband*innen samt Teilnahmeerklärung. Am darauffolgenden Tag werden die Patient*innen kontaktiert und gefragt, ob sie an der Studie teilnehmen möchten oder nicht.

Die Patient*innen werden mündlich und schriftlich darüber aufgeklärt, dass ihre Teilnahme an der Studie freiwillig, kostenfrei und ohne Entschädigung/ Entlohnung erfolgt. Es wird außerdem informiert, dass bei persönlichem Wunsch der Patient*innen die Einwilligung zur Studienteilnahme jederzeit ohne Angabe von Gründen widerrufen werden kann, ohne dass ihnen dadurch Nachteile entstehen.

Alle Patient*innen die an der Studie teilnehmen wollen, müssen einwilligungsfähig sein. Die Einwilligungsfähigkeit der Proband*innen ist ein ausdrückliches Einschlusskriterium dieser Studie. Nicht einwilligungsfähige Personen sind von der Teilnahme ausgeschlossen.

#### 4.4.2. Aufklärung

Alle Proband*innen werden in vollem Umfang und in verständlicher Form über Ablauf und Auswertung informiert. Die Proband*innen und wenn von den Proband*innen gewünscht, auch ihre Angehörigen bzw. gesetzlichen Betreuer/gewillkürten Vertreter werden mündlich und schriftlich über die Inhalte der Studie, insbesondere über die Untersuchungsmethoden und die Interventionen sowie ihr Widerrufsrecht umfassend aufgeklärt und über die Gewährleistung des Datenschutzes informiert. Sie erhalten zudem eine ausführliche Teilnehmerinformation.

#### 4.4.3. Einverständnis zur Studienteilnahme

Die interessierten Patient*innen werden im persönlichen Gespräch zwischen Proband*in und Studienleitung sowie schriftlich über die Motivation, die Ziele und den Ablauf des geplanten Studienvorhabens informiert. Die Patient*innen erhalten im Aufklärungsgespräch die Teilnehmerinformationen zur Studie zusammen mit der Einwilligungserklärung in schriftlicher Form. Die Proband*innen unterschreiben anschließend eine Einverständniserklärung für die informierte, freiwillige Einwilligung in die Studie. Die Proband*innen erhalten eine Kopie der Einwilligungserklärung für ihre persönlichen Unterlagen.

Sollte eine Erweiterung zum jetzt beantragten Vorhaben notwendig werden, werden diese der Ethikkommission in einem Amendement zur erneuten Bescheidung vorgelegt. Sollten andere Änderungen notwendig werden, wird neben der Ethikkommission, die von den Änderungen betroffenen Abteilungen (z.B. Datenschützer*innen) hinzugezogen. Sollte die Ethikkommission empfehlen, die Patient*innen über diese Ergänzung ebenfalls zu unterrichten, werden wir dies in Abstimmung (bzgl. der Form) mit der Ethikkommission tun.

## Art der Prüfung

Es handelt sich um ein Humanexperiment mit sonstiger Prüfung (neurowissenschaftliches/ neuropsychologisches Experiment)

## Gesetzliche Bestimmung und Verordnungen

Betrifft CMill mit virtueller Realität (VR):

Das Laufband C-Mill +VR by Motek (DIH GmbH, Köln, GER) ist ein Medizinprodukt, das für den Einsatz in der Therapie vorgesehen ist. Wie für die Anwendung von Medizinprodukten im Rahmen der Therapie vorgesehen, werden die Bestimmungen der Medizinprodukte-Betreiberverordnung, kurz MPBetreibV (Version vom 27.092016), beachtet.

Alle Behandler*innen in der Studie werden von berechtigten Einweiser*innen (Peter Wendland, Leiter Physiotherapie oder Timo Mihm, stellv. Leiter Physiotherapie) in die Nutzung des Laufbandes nach den Vorgaben der §5 Abs. 1 MPBetreibV eingewiesen. Für die Behandler*innen sind dies Routineabläufe.

Außerdem besteht ein enger Austausch zur Firma Motek.

Die weitere Therapie in beiden Gruppen entspricht dem etablierten Standard der Rehabilitation im NRZ „Godeshöhe“. Hier findet kein weiteres studienbedingtes Eingreifen in die Therapieabläufe statt, so dass hier keine zusätzlichen Bestimmungen und Verordnungen gelten.

**Für das gesamte Vorhaben** gelten Datenschutzbestimmungen, die unter Punkt B., 12 detailliert ausgeführt werden.

## Welche Vorprüfungen sind durchgeführt worden

Das CMill + VR wird bereits im klinischen und wissenschaftlichen Kontext eingesetzt. Nachfolgend wird der derzeitige Erkenntnisstand, eigene Vorarbeiten und relevante Literatur sowie fortbestehender Forschungsbedarf aufgezeigt.

### 7.1. Derzeitiger Erkenntnisstand zur Beeinträchtigung der Gehfähigkeit durch das Tragen eines MNS

Es ist bekannt, dass jegliche Masken zur Bedeckung von Mund und Nase, unabhängig ob medizinisch oder nicht-medizinisch, einen wesentlichen Teil des Gesichts bedecken. Insbesondere die FFP2-Masken ragen aufgrund ihrer Form durchaus in das untere Gesichtsfeld hinein (Kal et al., 2020a, Klatt et al., 2021). Es ist daher denkbar, dass dies eine Gesichtsfeldbeeinträchtigung bedingt, die einen Einfluss auf die visuelle Wahrnehmung und somit auf die Anpassungsfähigkeit des Ganges hat (Buckley et al., 2011; Rietdyk & Rhea, 2011), denn das untere Gesichtsfeldes liefert wichtige Informationen für eventuell notwendige Ganganpassungen (z.B. das Ausweichen oder Übersteigen von Hindernissen) und sicheres Gehen (Buckley et al., 2011). Eine Einschränkung in diesem Bereich durch das Tragen eines MNS könnte zu einer Beeinträchtigung der Ganganpassungsfähigkeit und damit möglicherweise zu einem gesteigerten Stolper- und Sturzrisiko, insbesondere unter den älteren und neurologisch Vorerkrankten Menschen, beitragen (Klatt et al., 2021; Callisaya et al., 2020; Rietdyk & Rhea, 2011; Lord et al., 2002). Bei diesen Menschen können u.a. Gleichgewichts-, Geh- und Reaktionsfähigkeit bereits beeinträchtigt sein, so dass die zusätzlichen Einschränkungen des Gesichtsfeldes durch das Tragen des MNS und die damit verbundene reduzierte sensorische Information ggf. nicht mehr so leicht kompensiert werden können und sich somit das Stolper- und Sturzrisiko massiv erhöhen könnte (Klatt et al., 2021; Kal et al., 2020a; Yakubovich et al., 2020; Callisaya et al., 2020).

Eine erste wissenschaftliche Debatte ist darüber entstanden, was betroffenen Menschen geraten werden könnte, um ein vermeintlich erhöhtes Sturzrisiko zu kompensieren. Allerdings wird aus der theoriebasierten Diskussion deutlich, dass eindeutige und personalisierte Empfehlungen derzeit schwierig sind. Hierfür fehlt auch die tatsächliche praktische Untersuchung, die Prävalenzen und eingeschränkte Funktionen bei Betroffenen aufzeigen würde und von der sich erste Empfehlungen ableiten ließen. Auch edukative Ansätze könnten relevant sein, für derartig edukative Ansätze ist es jedoch relevant zu wissen, wer zu den Betroffenen tatsächlich zählt und welche Aspekte der Ganganpassungsfähigkeit betroffen sind. Hierfür ist es relevant, diese Frage nicht nur theoretisch zu erörtern, sondern tatsächliche Prävalenzen beispielsweise in der neurologischen Population zu erheben.

### 7.2. Eigene Untersuchungen und relevante Literatur

eigene Arbeit des Kooperationspartners: McCrum, 2020

Literaturangaben: Klatt et al., 2021; Kal et al., 2020a; 2020b; Yakubovich et al., 2020; Callisaya et al., 2020

### 7.3. Fortbestehender Forschungsbedarf

1. Klärung, ob es in der Kohorte neurologisch vorerkrankter Menschen tatsächlich einen Unterschied in der Ganganpassungsfähigkeit macht, ob ein MNS getragen wir oder nicht.
2. Zu verstehen, wie Betroffene ihre Gehfähigkeit mit und ohne MNS subjektiv einschätzen.
3. Welche Aspekte der Ganganpassungsfähigkeit am ehesten durch das Tragen eines MNS beeinträchtigt werden.
4. Zu untersuchen, ob das Tragen eines MNS evtl. auch eine Auswirkung auf die Ergebnisse üblicher, klinischer Mobilitätsassessments hat.
5. Empfehlungen für Betroffene und Kliniker*innen abzuleiten.

## Pharmakologisch-toxikologische Prüfung

Entfällt

## Mögliche Komplikationen oder Risiken

Bei der Durchführung der geplanten Untersuchungen ist kein gesteigertes Risiko durch die Studienteilnahme zu erwarten. Auch werden keine Komplikationen in der Durchführung und Folgeprobleme für die Proband*innen und Angehörigen erwartet.

Allerdings ist die neurologische Patientenpopulation, mit der im Rahmen dieser Studie gearbeitet werden soll, möglicherweise in ihrer körperlichen und geistigen Leistungsfähigkeit eingeschränkt, was zu verschiedenen krankheitsbezogenen Risiken führen könnte, z.B. Gleichgewichtsverlust mit Stürzen, Schwierigkeiten in der Orientierung, Kreislaufprobleme, Stimmungsschwankungen, Medikamentenfluktuationen, etc. Es besteht hier jedoch kein erhöhtes Risiko durch die Studienteilnahme. Die Patient*innen werden von erfahrenen Therapeut*innen der Neurorehabilitation betreut, die sich mit dem Krankheitsbildern auskennen, mögliche Risiken einschätzen und abwenden können sowie den Umgang mit den Trainingsgeräten betreuen.

Des Weiteren hat das C-Mill by Motek extra ein Gurtsicherungssystem für Patient*innen, sowie Geländer auf beiden Seiten, einen Not-Aus-Schalter und eine Lichtschranke, die verhindert das Patient*innen zu weit Richtung Ende des Laufbandes kommen. Diese Vorrichtungen dienen der Sicherheit der Patient*innen. Alle Patient*innen werden in Einzeltherapien betreut, so dass sie im direkten Austausch mit erfahrenen Therapeut*innen stehen.

## Nutzen-Risiko-Abwägung

Die Maßnahmen finden im Rahmen der stationären, neurologischen Rehabilitation statt. Nebenwirkungen, die ausschließlich in den angewandten Testverfahren liegen, sind nicht zu erwarten. Die Patient*innen werden durchgehend von erfahrenen Therapeut*innen/ Tester*innen angeleitet und beobachtet. Eine zu starke Belastung ist dabei nicht zu erwarten und würde durch die jeweiligen Therapeut*innen/ Tester*innen unterbunden.

Könnte durch diese Studie gezeigt werden, dass das Tragen eines MNS einen Einfluss auf die Gangsicherheit von Menschen mit einer neurologischen Erkrankung hat, wäre dies eine wichtige Erkenntnis für die Betroffenen sowie für diejenigen, die im Versorgungskontext mit den betroffenen Menschen arbeiten, um ihnen Hilfen anzubieten und die Versorgung und/ oder Therapie entsprechend anzupassen (z.B. Gangtraining mit MNS durchzuführen).

Außerdem könnten diese Erkenntnisse als Grundlage genutzt werden, um mögliche Kompensationsstrategien zu erarbeiten. Denn auch wenn die CoVID-19 Pandemie zu Ende gehen sollte und das Tragen eines MNS nicht länger nötig ist, gibt es im klinischen Umfeld immer wieder Situationen, wo neurologische Patient*innen ggf. einen MNS tragen müssen. Auch weitere Infektionsgeschehen können zukünftig nicht ausgeschlossen werden und auch hierfür könnten diese Erkenntnisse nützlich sein. So lange die CoVID-19 Pandemie jedoch andauert ist die Sturzprophylaxe, zu der auch die Gefährdungseinschätzung für bestimmte Gruppen gehört, zur Entlastung des Gesundheitssystems höchst relevant.

## Zwischenauswertung und Abbruchkriterien

Es ist keine Zwischenauswertung geplant.

Bei unerwarteten unerwünschten Nebenwirkungen, von denen jedoch nicht auszugehen ist, wird die Studie abgebrochen.

Bei persönlichem Wunsch der Patient*innen kann der Abbruch jederzeit ohne Angabe von Gründen erfolgen und ohne das den Betroffenen daraus ein Nachteil entsteht.

## Datenquellen, Datenhaltung und Datenschutz

### 12.1. Datenquelle

Es werden keine bereits vorhandenen Daten ausgewertet, sondern motorische, soziodemographische und gesundheitsbezogene/ klinische Daten werden neu erhoben. Für eine detaillierte Auskunft über die einzelnen Daten sei auf „2.2. Assessments“ verwiesen.

### 12.2. Datenhaltende Stelle

NRZ „Godeshöhe“ e. V.

Waldstraße 2-10 ***∙*** 53177 Bonn

Alle eingeschlossenen Patient*innen werden in einer elektronischen Akte (electronic Case Report Form, eCRF) erfasst, die von den an der Studie beteiligten Wissenschaftler*innen geführt wird. Alle, die Patient*innen betreffenden Daten werden entweder direkt digital erfasst oder von Papierversionen (Kopien der motorischen (Routine-)Testungen und Fragebögen) übertragen. Die Studiendaten werden mittels der etablierten Datenmanagementplattform Castor (https://www.castoredc.com/) erfasst. Diese Datenmanagementplattform erfüllt die Richtlinien für Informationssicherheit (ISO 27001; https://www.castoredc.com/security-statement/), die regelmäßig den aktuellsten Anforderungen entsprechend angepasst werden. Die Datenserver entsprechen geltenden Richtlinien (ISO27001, ISO9001) sowie nationalen oder internationalen Standards (HIPPA, NEN7510). Während der Datenruhe sind die dort gespeicherten Daten verschlüsselt. Während der Datenbearbeitung werden diese entschlüsselt, wobei hier verschiedenen Decodierungslevel festgelegt werden können. Jede*r Benutzer*in erhält ein individuelles, passwortgeschütztes Benutzerkonto über das die Daten verwaltet werden. Wer Zugang zu den Daten erhält legt die*er jeweilige Studienleiter*in fest. Die erhobenen Daten bleiben Eigentum der Daten haltenden Stellen (s.o.).

Für die Datenverarbeitung sind folgende Personen zuständig:

Mareike Eschweiler, MSc.

Leitung Therapiewissenschaften

NRZ „Godeshöhe“ e.V.

Waldstraße 2-10

53177 Bonn

Telefon +49 228 381 – 559

E-Mail: [m.eschweiler@godeshoehe.de](mailto:m.eschweiler@godeshoehe.de)

Dipl. Psych. Jochen Saliger

Leitung kognitive Rehabilitation

NRZ „Godeshöhe“ e.V.

Waldstraße 2-10

53177 Bonn

Telefon +49 228 381 – 702

E-Mail: j.saliger@godeshoehe.de

Für Datenschutz-Belange ist folgende Datenschutzbeauftragte außerdem zuständig und für Proband*innen ansprechbar:

NRZ „Godeshöhe“ e.V.

Datenschutzbeauftragte

Waldstraße 2-10

53177 Bonn

Telefon: +49 228 381-643

E-Mail: datenschutz@godeshoehe.de

### 12.3. Datenschutz

#### 12.3.1. Positive Stellungnahme der Datenschutzbeauftragten

Die Datenschutzbeauftragte des NRZ „Godeshöhe“ e.V. hat den Ethikantrag sowie die Teilnahmeerklärung für Studienteilnehmer*innen zugeleitet bekommen, datenschutzrechtlich geprüft und ggf. nach Korrektur freigegeben.

Außerdem ist die Beachtung der Datenschutz-Grundverordnung (DSGVO), des Bundesdatenschutzgesetzes (BDSG), des Gesundheitsdatenschutzgesetzes NRW und die revidierte Deklaration von Helsinki in der aktuellen Fassung von Fortaleza (Brasilien), 2013 in vollem Umfang sichergestellt.

#### 12.3.2. Auswertung mit (faktisch) anonymisierten Daten

Die Datenerfassung in Castor erfolgt pseudonymisiert mittels der Studien-ID (s. Punkt 11.3.3). Die Protokollbögen der Testungen sowie Kopien der Protokollbögen der motorischen (Routine-)Testung und die Fragebögen werden unmittelbar pseudonymisiert, so dass die weitere Datenverarbeitung faktisch anonymisiert erfolgt.

Es gibt eine Schlüsselliste im NRZ für die dort eingeschlossenen Teilnehmer*innen. Die Liste gibt Aufschluss über die Zugehörigkeit der Probanden-IDs zu den einzelnen Studienteilnehmer*innen. Diese Liste wird bei von Frau Eschweiler aufbewahrt. Die Aufbewahrung der Schlüsselliste erfolgt in einem abschließbaren Schrank; allerdings unabhängig von den Protokollbögen. Die Aufbewahrung einer solchen Schlüsselliste ist durch die Auskunftspflicht gegenüber den Proband*innen begründet. Nach Abschluss der Datenerhebung wird diese Schlüsselliste umgehend vernichtet.

#### 12.3.3. Verhinderung der Personenidentifizierung

Alle Proband*innen, die an der Studie teilnehmen erhalten eine ID, die sich aus Buchstaben und Zahlen zusammensetzt. Die Buchstaben sind für jede*n Proband*in gleich und greifen ein Studienkürzel, CMill-NMS für C-Mill by Motek mit/ ohne MNS, auf. Die Zahl kommt nur einmal vor und bezieht sich auf die Patientenzahl. Sie kann folglich von 001 bis 50 reichen. Die Nummerierung der Patient*innen erfolgt aufsteigend.

#### 11.3.4. Zugänglichkeit der Daten für Dritte

Zugriff auf die pseudonymisierten Studiendaten haben nur unmittelbar an der Studie beteiligte, zur Verschwiegenheit verpflichtete Mitarbeiter*innen des NRZ „Godeshöhe“ sowie Dr. Giannouli und PD Dr. McCrum. Allerdings ist geplant, nach Abschluss der Studie die anonymisierten Daten anderen Wissenschaftler*innen im Sinne einer „open data availability“ zugänglich zu machen. Dies entspricht den Anforderungen der geplanten Präregistrierung und ist heutzutage ein Qualitätskriterium nachvollziehbarer wissenschaftlicher Praxis. Die anonymisierten Daten werden in einem Portal, speziell für öffentliche, wissenschaftliche Datensätze zur Verfügung gestellt, z.B. Science Framework oder Zenodo. Hier folgen wir gerne auch alternativen Empfehlungen durch die Ethikkommission.

Des Weiteren wird mit den Rohdaten folgendermaßen verfahren: Die soziodemografischen und klinischen Daten werden direkt im Datenmanagementsystem Castor, anonymisiert erfasst. Die Protokollbögen der Testungen und die Fragebögen aller Studienteilnehmer*innen werden pseudonymisiert in abschließbaren Schränken im Büro von Frau Eschweiler für die Zeit der Datenerhebung aufbewahrt und gemäß den Maßgaben der guten wissenschaftlichen Praxis (GPS) im NRZ „Godeshöhe“ archiviert. Eine Vernichtung der Unterlagen erfolgt entsprechend der GPS nach zehn Jahren. Es erfolgt keine Weitergabe des Materials an Dritte.

Alle Studiendaten werden sukzessive, parallel zur laufenden Rekrutierung, in der Datenmanagementplattform Castor erfasst (s. Punkt 11.2), die, wie oben beschrieben, die relevanten Vorschriften zur Datensicherheit erfüllt. Darüber hinaus werden keine Dokumente mit personen- bzw. gesundheitsbezogenen Informationen in Systemen der Online-Datensicherung (z.B. Dropbox, Google Drive, iCloud) gespeichert.

Die spätere Publikation der Ergebnisse in internationalen Fachzeitschriften oder die Vorstellung auf Kongressen erfolgt in vollständig anonymisierter Form. Ein Personenbezug wird nicht möglich sein.

Alle an der Studie beteiligten Personen sind zur Verschwiegenheit verpflichtet. Die Beachtung der Datenschutz-Grundverordnung (DSGVO), des Bundesdatenschutzgesetzes (BDSG), des Gesundheitsdatenschutzgesetzes NRW und die revidierte Deklaration von Helsinki in der aktuellen Fassung von Fortaleza (Brasilien), 2013 wird in vollem Umfang sichergestellt.

Die Patient*innen erhalten ausführliche Informationen über die Verarbeitung Ihrer Daten und die für die Datenverarbeitung verantwortlichen Personen sowie die Kontaktinformationen der Datenschutzbeauftragten des NRZ sowie der Datenschutz-Aufsichtsbehörde des Landes NRW. Zusätzlich wird betont, dass die Patient*innen jederzeit das Recht haben, Einsicht in ihre Daten zu nehmen, die während der Studie erhoben werden, und ggf. eine Löschung zu verlangen.

## Probandenaufklärung und Einwilligung

Alle Studienteilnehmer*innen werden in vollem Umfang und in verständlicher Form über Ablauf und Auswertung informiert. Die Studienteilnehmer*innen und wenn von den Studienteilnehmer*innen gewünscht, auch ihre Angehörigen werden mündlich und schriftlich über die Inhalte der Studie, insbesondere über die Untersuchungsmethoden sowie ihr Widerrufsrecht umfassend aufgeklärt und über die Gewährleistung des Datenschutzes informiert. Sie erhalten zudem eine ausführliche Teilnehmerinformation (s. Anlage I).

Die interessierten Patient*innen werden im persönlichen Gespräch zwischen Studienteilnehmer*in und Studienleitung sowie schriftlich über die Motivation, die Ziele und den Ablauf des geplanten Studienvorhabens informiert. Die Patient*innen erhalten im Aufklärungsgespräch die Teilnehmerinformationen zur Studie zusammen mit der Einwilligungserklärung (s. Anlage I) in schriftlicher Form. Die Studienteilnehmer*innen unterschreiben anschließend eine Einverständniserklärung für die informierte, freiwillige Einwilligung in die Studie. Die Studienteilnehmer*innen erhalten eine Kopie der Einwilligungserklärung für ihre persönlichen Unterlagen. Die informierte, schriftliche Einwilligung bildet die Rechtsgrundlage für die angestrebte Datenerhebung und -verarbeitung im Rahmen der Studie (vgl. Artikel 6 Nr. 1 DSGVO).

Sollte eine Erweiterung zum jetzt beantragten Vorhaben notwendig werden, werden diese der Ethikkommission in einem Amendement zur erneuten Bescheidung vorgelegt. Sollten andere Änderungen notwendig werden, wird neben der Ethikkommission, die von den Änderungen betroffenen Abteilungen (z.B. Datenschützer) hinzugezogen. Sollte die Ethikkommission empfehlen, die Patient*innen über diese Ergänzung ebenfalls zu unterrichten, werden wir dies in Abstimmung (bzgl. der Form) mit der Ethikkommission tun.

## Versicherungsschutz

## Es wird keine zusätzliche Versicherung für Proband*innen abgeschlossen. Allerdings könnten Patient*innen, bei einer schulhaft verursachten Schädigung, einen Entschädigungsanspruch geltend machen, welcher über die Haftpflichtversicherung des Rehabilitationszentrums abgedeckt wäre.

# C**. PATIENTENINFORMATION UND EINVERSTÄNDNISERKLÄRUNG**

s. Anlage I

# D. UNTERSCHRIFTEN UND DATUM

Hiermit erklären wir unser Einverständnis zur Durchführung der Studie: „Einfluss des Tragens eines Mund-Nasen-Schutzes auf die Ganganpassungsfähigkeit von Menschen mit einer neurologischen Erkrankung“ im NRZ „Godeshöhe“, Bonn- Bad Godesberg.

Mareike Eschweiler, MSc.

Leitung Therapiewissenschaften

Prof. Dr. med. Hans Karbe

Ärztlicher Direktor des Neurologischen Rehabilitationszentrum Godeshöhe, e.V.

Klaus Köhring

Klinikdirektor des Neurologischen Rehabilitationszentrum Godeshöhe, e.V.

**Datum:** Bonn- Bad Godesberg, den 22.06.2021

# Literatur

Beghi, E., Gervasoni, E., Pupillo, E., Bianchi, E., Montesano, A., Aprile, I., Agostini, M., Rovaris, M., Cattaneo, D., & NEUROFALL Group (2018). Prediction of Falls in Subjects Suffering From Parkinson Disease, Multiple Sclerosis, and Stroke. Archives of physical medicine and rehabilitation, 99(4), 641–651. https://doi.org/10.1016/j.apmr.2017.10.009

Buckley, J. G., Timmis, M. A., Scally, A. J., & Elliott, D. B. (2011). When is visual information used to control locomotion when descending a kerb?. PloS one, 6(4), e19079. https://doi.org/10.1371/journal.pone.0019079

Bundesregierung (2021d). Videokonferenz der Bundeskanzlerin mit den Regierungschefinnen und Regierungschefs der Länder am 22. März 2021-BESCHLUSS. Available at: https://www.bundesregierung.de/resource/blob/997532/1879672/2854753dbc7549432db7f0bba94e8c0f/2021-03-22-mpk-data.pdf?download=1, last access 23.03.2021

Bundesregierung (2021c). So schützen uns Masken im Alltag. Available at: https://www.bundesregierung.de/breg-de/aktuelles/regelung-zu-masken-1842704, last access 19.03.2021

Bundesregierung. (2021b). Videoschaltkonferenz der Bundeskanzlerin mit den Regierungschefinnen und Regierungschefs der Länder am 10. Februar 2021-Beschluss. Available at: https://www.bundesregierung.de/resource/blob/975226/1852514/508d851535b4a599c27cf320d8ab69e0/2021-02-10-mpk-data.pdf?download=1, last access: 19.03.2021

Bundesregierung. (2021a). Videoschaltkonferenz der Bundeskanzlerin mit den Regierungschefinnen und Regierungschefs der Länder am 19. Januar 2021-Beschluss. Available at: https://www.bundesregierung.de/resource/blob/975226/1840868/1c68fcd2008b53cf12691162bf20626f/2021-01-19-mpk-data.pdf?download=1, last access: 19.03.2021

Bundesregierung. (2020j). Videoschaltkonferenz der Bundeskanzlerin mit den Regierungschefinnen und Regierungschefs der Länder am 25. November 2020-Beschluss. Available at: https://www.bundesregierung.de/resource/blob/975226/1820174/fd9794fa8b8e0ec555f005677509c242/2020-11-25-mpk-beschluss-data.pdf?download=1, last access: 19.03.2021

Bundesregierung. (2020i). Videokonferenz der Bundeskanzlerin mit den Regierungschefinnen und Regierungschefs der Länder am 28. Oktober 2020-Beschluss. Available at: https://www.bundesregierung.de/resource/blob/975226/1805024/5353edede6c0125ebe5b5166504dfd79/2020-10-28-mpk-beschluss-corona-data.pdf?download=1, last access: 19.03.2021

Bundesregierung. (2020h). Telefonschaltkonferenz des Chefs des Bundeskanzleramts mit den Chefinnen und Chefs der Staats- und Senatskanzleien der Länder am 7.Oktober 2020-Beschluss. Available at: https://www.bundesregierung.de/resource/blob/975228/1796766/65f8f7a714ddb688953c4c0c6790dcd8/2020-10-07-beschluss-cds-corona-data.pdf?download=1, last access: 19.03.2021

Bundesregierung. (2020g). Videoschaltkonferenz der Bundeskanzlerin mit den Regierungschefinnen und Regierungschefs der Länder am 29.September 2020-Beschluss. Avaliable at: https://www.bundesregierung.de/resource/blob/975226/1792238/bbe262252712bf09bbb85f93effa9b15/2020-08-29-beschluss-mpk-data.pdf?download=1, last access: 19.03.2021

Bundesregierung. (2020f). Telefonschaltkonferenz der Bundeskanzlerin mit den Regierungschefinnen und Regierungschefs der Länder am 27. August 2020-Beschluss. Available at: https://www.bundesregierung.de/resource/blob/975226/1780568/2f9c77a8e8a549bcac8123fbeff4ee27/2020-08-27-beschluss-mpk-data.pdf?download=1, last access: 19.03.2021

Bundesregierung (2020e). Die „AHA-Regeln“ im neuen Alltag. Available at: https://www.bundesregierung.de/breg-de/themen/coronavirus/die-aha-regeln-im-neuen-alltag-1758514, last access 19.03.2021

Bundesregierung. (2020d). Telefonschaltkonferenz der Bundeskanzlerin mit den Regierungschefinnen und Regierungschefs der Länder am 15. April 2020-Beschluss. Available at: https://www.bundesregierung.de/breg-de/themen/coronavirus/bund-laender-beschluss-1744224, last access: 19.03.2021

Bundesregierung. (2020c). Besprechung der Bundeskanzlerin mit den Regierungschefinnen und Regierungschefs der Länder vom 22.03.2020-Beschluss. Available at: https://www.bundesregierung.de/breg-de/themen/coronavirus/besprechung-der-bundeskanzlerin-mit-den-regierungschefinnen-und-regierungschefs-der-laender-vom-22-03-2020-1733248, last access: 19.03.2021

Bundesregierung (2020b). Leitlinien zum Kampf gegen die Corona-Epidemie vom 16.03.2020. Available at: https://www.bundesregierung.de/breg-de/themen/coronavirus/leitlinien-zum-kampf-gegen-die-corona-epidemie-vom-16-03-2020-1730942, last access, 19.03.2021

Bundesregierung (2020a). Besprechung der Bundeskanzlerin mit den Regierungschefinnen und Regierungschefs der Länder am 12. März 2020-Beschluss. Available at: https://www.bundesregierung.de/breg-de/themen/coronavirus/beschluss-zu-corona-1730292, last access 19.03.2021

Callisaya, M., Hill, K., Hill, A.M., Mackintosh, S., Batchelor, F., Said, C.M., Sherrington, C., Dawson, R., West, C., & Fu, S. (2020). Rapid Response: Face masks and risk of falls – a vision for personalised advice and timing? Available from: https://www.bmj.com/content/371/bmj.m4133/rr, last access 25.02.2021

Dias, N., Kempen, G. I., Todd, C. J., Beyer, N., Freiberger, E., Piot-Ziegler, C., Yardley, L., & Hauer, K. (2006). Die Deutsche Version der Falls Efficacy Scale-International Version (FES-I) [The German version of the Falls Efficacy Scale-International Version (FES-I)]. Zeitschrift fur Gerontologie und Geriatrie, 39(4), 297–300. https://doi.org/10.1007/s00391-006-0400-8

Freund, A. (2021). Is the end of the coronavirus pandemic in sight? Available at: https://www.dw.com/en/is-the-end-of-the-coronavirus-pandemic-in-sight/a-56740737, last access 19.03.2021

Guralnik, J. M., Ferrucci, L., Pieper, C. F., Leveille, S. G., Markides, K. S., Ostir, G. V., Studenski, S., Berkman, L. F., & Wallace, R. B. (2000). Lower extremity function and subsequent disability: consistency across studies, predictive models, and value of gait speed alone compared with the short physical performance battery. The journals of gerontology. Series A, Biological sciences and medical sciences, 55(4), M221–M231. <https://doi.org/10.1093/gerona/55.4.m221>

Hoehn, M.M., Yahr, M.D. (1967) Parkinsonism: onset, progression and mortality. In: Neurology. 17(5), 427- 442

Hofheinz, M., Schusterschitz, C., & Mehrholz, J. (2011). Der Timed Up and Go-Test mit motorischer und kognitiver Zusatzaufgabe – ein geeigneter Test zur Einschätzung der Sturzgefahr? HeilberufeSCIENCE, 2 (1): 31–37. DOI 10.1007/s16024-011-0108-6

Hoogkamer, W., Bruijn, S. M., Potocanac, Z., Van Calenbergh, F., Swinnen, S. P., & Duysens, J. (2015). Gait asymmetry during early split-belt walking is related to perception of belt speed difference. Journal of neurophysiology, 114(3), 1705–1712. https://doi.org/10.1152/jn.00937.2014

Hunter, S. W., Divine, A., Frengopoulos, C., & Montero Odasso, M. (2018). A framework for secondary cognitive and motor tasks in dual-task gait testing in people with mild cognitive impairment. BMC geriatrics, 18(1), 202. https://doi.org/10.1186/s12877-018-0894-0

Kal, E. C., Young, W. R., & Ellmers, T. J. (2020a). Face masks, vision, and risk of falls. BMJ (Clinical research ed.), 371, m4133. https://doi.org/10.1136/bmj.m4133

Kal, E. C., Young, W. R., & Ellmers, T. J. (2020b). Rapid Response: Authors’ response to: “Face masks and risk of falls – a vision for personalised advice and timing?” by Callisaya et al. Available from: https://www.bmj.com/content/371/bmj.m4133/rr-1, last access 25.02.2021

Klatt, B. N., & Anson, E. R. (2021). Navigating Through a COVID-19 World: Avoiding Obstacles. Journal of neurologic physical therapy : JNPT, 45(1), 36–40. https://doi.org/10.1097/NPT.0000000000000338

Kurtzke J. F. (1983). Rating neurologic impairment in multiple sclerosis: an expanded disability status scale (EDSS). Neurology, 33(11), 1444–1452. https://doi.org/10.1212/wnl.33.11.1444

Land NRW (2021). Verordnung zum Schutz vor Neuinfizierungen mit dem Coronavirus SARS-CoV-2 (Coronaschutzverordnung – CoronaSchVO). Available at: https://www.land.nrw/sites/default/files/asset/document/2021-03-05_coronaschvo_ab_08.03.2021_lesefassung.pdf

Lord, S. R., Dayhew, J., & Howland, A. (2002). Multifocal glasses impair edge-contrast sensitivity and depth perception and increase the risk of falls in older people. Journal of the American Geriatrics Society, 50(11), 1760–1766. https://doi.org/10.1046/j.1532-5415.2002.50502.x

Mathias, S., Nayak, U. S., & Isaacs, B. (1986). Balance in elderly patients: the "get-up and go" test. Archives of physical medicine and rehabilitation, 67(6), 387–389.

McCrum, C. (2020). Rapid Response: Walking slower increases anterior stability to a trip: a consideration for face masks and falls risk. Available from: https://www.bmj.com/content/371/bmj.m4133/rr-0, last access 25.02.2021

Nightingale, E. J., Pourkazemi, F., & Hiller, C. E. (2014). Systematic review of timed stair tests. Journal of rehabilitation research and development, 51(3), 335–350. https://doi.org/10.1682/JRRD.2013.06.0148

Rapp, K., Freiberger, E., Todd, C., Klenk, J., Becker, C., Denkinger, M., Scheidt-Nave, C., & Fuchs, J. (2014). Fall incidence in Germany: results of two population-based studies, and comparison of retrospective and prospective falls data collection methods. BMC geriatrics, 14, 105. https://doi.org/10.1186/1471-2318-14-105

Rietdyk, S., & Rhea, C. K. (2011). The effect of the visual characteristics of obstacles on risk of tripping and gait parameters during locomotion. Ophthalmic & physiological optics : the journal of the British College of Ophthalmic Opticians (Optometrists), 31(3), 302–310. https://doi.org/10.1111/j.1475-1313.2011.00837.x

Robert-Koch-Institut (RKI). (2021): Coid-19: Fallzahlen und Deutschland und weltweit. Available at: https://www.rki.de/DE/Content/InfAZ/N/Neuartiges_Coronavirus/Fallzahlen.html, last access 19.03.2021

Rosengren, K. S., McAuley, E., & Mihalko, S. L. (1998). Gait adjustments in older adults: activity and efficacy influences. Psychology and aging, 13(3), 375–386. https://doi.org/10.1037//0882-7974.13.3.375

Timmermans, C., Roerdink, M., Janssen, T.W.J., Beek, P.J., & Meskers, CGM. (2019). Automatized, standardized, and patient-tailored progressive walking-adaptability training: a proof-of-concept study. Physical Therapy, 99: 882–891.

United Nations, UN (2021). Pandemic will not end for anyone, ‘until it ends for everyone’. Available at: https://news.un.org/en/story/2021/01/1082762, last access 19.03.2021

World Health Organization, WHO (2021). Coronavirus disease (COVID-19) Weekly Epidemiological Update and Weekly Operational Update. Available at: https://www.who.int/emergencies/diseases/novel-coronavirus-2019/situation-reports, last access 19.03.2021

WHO (2020). Mask use in the context of COVID-19. Available at: file:///C:/Users/eschweilerm/Downloads/WHO-2019-nCov-IPC_Masks-2020.5-eng%20(2).pdf, last access: 23.03.2021

Yakubovich, S., Israeli-Korn, S., Halperin, O., Yahalom, G., Hassin-Baer, S., & Zaidel, A. (2020). Visual self-motion cues are impaired yet overweighted during visual-vestibular integration in Parkinson's disease. Brain communications, 2(1), fcaa035. https://doi.org/10.1093/braincomms/fcaa035

1. https://www.euro.who.int/en/health-topics/disease-prevention/nutrition/a-healthy-lifestyle/body-mass-index-bmi [↑](#footnote-ref-1)
