# Supplementary material for: A registered report of a crossover study on the effects of face masks on walking adaptability in people with Parkinson’s disease and multiple sclerosis
Source: PLoS One. 2023 Jun 29;18(6):e0286402. doi: 10.1371/journal.pone.0286402 (PMC10309975; doi:10.1371/journal.pone.0286402)
Supplement: S2 File — (DOCX) [file pone.0286402.s005.docx]

**Influence of wearing a facemask on gait adaptability in people with a neurological disorder.**

**Version 1, June 2021**

**Main contact:**

Mareike Eschweiler, MSc.

Head of Therapeutic Sciences

Neurological Rehabilitation Center "Godeshöhe" GmbH (NRZ "Godeshöhe")

Waldstraße 2-10

53177 Bonn

Phone +49 228 381-559

Fax +49 228 381-318

E-mail mareike.eschweiler@go.johanniter-kliniken.de

**Application for the granting of a vote by the Ethics Committee of the Medical Faculty of the University of Bonn**

To conduct a medical/scientific project that does not involve the clinical testing of a drug or medical device.

# A. FORMAL

## Project Name

Influence of wearing a facemask on gait adaptability in people with a neurological disorder.

## Applicants, responsible managers and cooperation partners:

### Applicant and responsible project manager:

Mareike Eschweiler, M.Sc.

Head of Therapeutic Sciences

NRZ "Godeshöhe" GmbH

Waldstraße 2-10

53177 Bonn

Phone +49 228 381 - 559

E-mail [mareike.eschweiler@go.johanniter-kliniken.de](mailto:mareike.eschweiler@go.johanniter-kliniken.de)

### Responsible Medical Director:

Prof. Dr. med. Hans Karbe

Medical Director

NRZ "Godeshöhe" GmbH

Academic lecturer for rehabilitation medicine

Faculty of Medicine

Rhenish Friedrich Wilhelm University Bonn

Waldstraße 2-10

53177 Bonn

Phone +49 228 381 - 206 (Secretariat: +49 228 381 - 207)

E-mail: [hans.karbe@go.johanniter-kliniken.de](mailto:hans.karbe@go.johanniter-kliniken.de);

(Secretariat: karbe.office@go.johanniter-kliniken.de)

I hereby declare my consent to conduct the study: "Influence of wearing a facemask on the gait adaptability of people with a neurological disease" at the NRZ "Godeshöhe", Bonn-Godesberg.

Prof. Dr. med. Hans Karbe

Medical Director of the NRZ "Godeshöhe

### Cooperation partner

Dr. Eleftheria Giannouli

University of Basel

Department of Sport, Exercise and Health

Department of Sports and Exercise Medicine

Birsstrasse 320 B

4052 Basel, Switzerland

Phone +41 61 207 47 45

E-mail eleftheria.giannouli@unibas.ch

Web <https://dsbg.unibas.ch>

Chris McCrum, PhD

Assistant Professor of Human Movement Sciences

Maastricht University

Department of Nutritional and Biological Sciences

Universiteitssingel 50

6229 ER Maastricht, Netherlands

E-mail chris.mccrum@maastrichtuniversity.nl

Web <https://www.maastrichtuniversity.nl/chris.mccrum>

The collaborations with Dr. Giannouli and Dr. McCrum consist of jointly developing the planned study. In the further course of the project, an exchange of data, analysis and analysis results as well as the joint preparation of manuscripts for publication purposes is planned.

Dr. McCrum has already contributed a paper to the scientific discourse on this topic in the BMJ (McCrum, 2020) and sees the clinical review as a relevant addition to the theoretical discussion to date.

There is already a close professional exchange with Dr. Giannouli for other study projects.

### Other persons involved

Dipl. Psych. Jochen Saliger

Management cognitive rehabilitation

NRZ "Godeshöhe" e.V.

Waldstraße 2-10

53177 Bonn

Phone +49 228 381-702

E-mail [jochen.saliger@go.johanniter-kliniken.de](mailto:jochen.saliger@go.johanniter-kliniken.de)

Peter Wendland

Physiotherapy management

NRZ "Godeshöhe" e.V.

Waldstraße 2-10

53177 Bonn

Phone +49 228 381-989

E-mail [peter.wendland@go.johanniter-kliniken.de](mailto:peter.wendland@go.johanniter-kliniken.de)

Florian Wolf, M.A.

Research assistant

Sports Therapist

NRZ "Godeshöhe" e.V.

Waldstraße 2-10

53177 Bonn

Phone +49 228 381 - 481

E-mail [florian.wolf@go.johanniter-kliniken.de](mailto:florian.wolf@go.johanniter-kliniken.de)

## Type and number of test centers

The planned study project will be conducted as a monocentric study.

There is only a professional cooperation as described above (2.3.) with Dr. Giannouli and PD Dr. McCrum.

## Cost unit

The study is financed from own funds of the NRZ "Godeshöhe".

In addition, an application for third-party funding was submitted to the Förderverein NRZ Bonn e.V., c/o NRZ Godeshöhe e.V., Waldstraße 2-10, 53177 Bonn, on May 30, 2021 (decision pending).

## Decision of the ethics committees in the same matter

No application has been made to any other ethics committee.

# B DESCRIPTION OF THE STUDY

## Scientific description of the project

### 1.1 State of research and scientific background

Since the beginning of 2020, the Covid-19 pandemic has repeatedly presented the world with new challenges (WHO, 2021; German government, 2020a; 2021). In particular, the containment of infection rates and the prevention of an overload of the health care system are the focus of all efforts (Bundesregierung, 2020a; 2020c). The measures taken to this end, which are constantly being adapted, affect the daily lives of many people (Bundesregierung, 2020a; 2020b; 2020c; 2020d; 2020e, 2020f; 2020g; 2020h; 2021d). Despite optimistic vaccination progress, the end of the pandemic as well as virus prevention measures is currently not foreseeable (UN, 2021; Freund, 2021; Callisaya et al., 2020). Repeatedly, new waves of infection occur (Bundesregierung, 2020h; 2021a; 2021d RKI, 2021), and with it, more stringent prevention measures (Bundesregierung, 2020i; 2021d).

In particular, the "distance hygiene everyday mask" rules (Bundesregierung, 2020d; 2020e; 2020f; WHO, 2020) and regular ventilation of enclosed spaces (Bundesregierung, 2020g) have proven to be an effective, individual contribution to containing the incidence of infection. The recommendation or regulation to wear everyday masks has now been replaced in many places by the obligation to wear a surgical or FFP2 mask (Bundesregierung, 2020d; 2020j; 2021a; 2021b; 2021c).

All masks, whether medical or non-medical, cover a significant portion of the face. Due to their shape, they definitely protrude into the lower visual field (Kal et al., 2020a, Klatt et al., 2021). It is conceivable that this visual field impairment has an impact on the perception of visual stimuli (Buckley et al., 2011; Rietdyk & Rhea, 2011), because this area of the lower visual field provides important information for any necessary gait adjustments (e.g., avoiding or stepping over obstacles) and safe walking (Buckley et al., 2011). Limitation in this area due to wearing a facemask could contribute to impaired gait performance and safety and possibly increased risk of trips and falls (Klatt et al., 2021; Callisaya et al., 2020; Rietdyk & Rhea, 2011; Lord et al., 2002). Kal and colleagues suggest that this may have an impact on gait safety in the elderly (Kal et al, 2020a). This assumption is supported by the study of Lord and colleagues (2002) in which they could show that already wearing multifocal glasses reduced gait safety. Older people, from the age of 65 years upwards, have an increased risk of falling (Rapp et al., 2014), so it is very likely that further restrictions, such as wearing a facemask, additionally increase the risk of falling. These negative consequences may be even more severe for the population of neurologically predisposed individuals (Klatt et al., 2021; Callisaya et al., 2020), who already have many times the increased risk of falls compared to the average population even without facemask (Beghi et al., 2018).

In people with an underlying neurological disorder, balance, walking, and reaction, among other abilities, are already impaired by the neurological disorders, so that the additional limitations of the visual field and the associated reduced sensory information may not be as easily compensated for by wearing a facemask, and thus the risk of tripping and falling could increase massively (Klatt et al., 2021; Kal et al., 2020a; Yakubovich et al., 2020; Callisaya et al., 2020).

An initial scientific debate has arisen about what affected people could be advised to do to compensate for a supposedly increased risk of falling. Kal et al. (2020a; 2020b) suggest a reduced walking pace for this purpose. They point out that the intuitive and sometimes advised downward gaze may have an additional negative impact on gait safety (Kal et al., 2020a). Callisaya et al, 2020 argue that a slowed walking pace could also reduce gait safety. McCrum (2020) points out that various aspects of postural control and mechanical stability are important and argues that reducing walking pace may be appropriate. He adds that this might even prevent falling forward (McCrum, 2020). Overall, it is clear from the discussion that explicit and personalized recommendations are currently difficult. There is also a lack of actual practical studies that would reveal prevalence and possibly initial limitations and from which initial recommendations could be derived. Therefore, the proposed study aims to investigate these aspects in a neurological cohort. Callisaya et al. (2020) also point out in their paper that education of potentially affected populations is an essential step. The research group states that knowledge of potential risk situations is an important prevention aspect. However, for such educational approaches, it is relevant to know who is actually among those affected. For this purpose, it is essential not only to discuss this question theoretically, but to survey actual prevalence, for example in the neurological population. For this, rather theory-guided scientific discussion leaves open the question of how prevalent the presumed phenomenon actually is in a cohort of neurologically ill people. Therefore, the planned study project will investigate whether "wearing a facemask" versus "not wearing a facemask" has an impact on gait and gait safety.

### 1.2 Study Objective:

In order to verify this, a standardized test to measure the adaptability of gait (CGait) on a VR-based treadmill (C-Mill+VR by Motek) once with and once without a mask as well as specific clinical tests (10 meter walk test, 10mWT; Timed Up and Go Test, TUG, TUG with dual task (TUG+DT) and climbing up and down stairs) will be performed in 50 people with a neurological disease. In addition, the participants will be asked to comment subjectively on their experience of walking with and without a mask using standardized questions.

This study can add clinical data for people with a neurological disease to the existing theoretical scientific discourse and thus make an important contribution to an existing scientific debate. In addition, these findings would serve the affected individuals in that it would allow for a scientifically based recognition of their additional limitations. In addition, following the demands of Callisaya and colleagues (2020) and Kal and colleagues (2020b), this could provide the basis for further clinical investigations regarding possible compensations or recommendations for affected individuals.

### 1.3 Questions and hypotheses:

The main question to be addressed in the planned study is whether wearing the facemask (FFP-2 mask) has an impact on the adaptability of gait (measured by the performance score (%) per CGait subtest for each of the two difficulty levels) in people with a neurological disease (*Question 1*). It is hypothesized that people with a neurological condition (Multiple Sclerosis or Parkinson's disease) will perform significantly worse on individual or all CGait subtests if they wear a facemask than if they do not (*Hypothesis 1*).

However, since clinically observed improvement cannot only be about standardized, objective measurements, the self-assessment of patients must also be given high priority. It should be clarified whether wearing a facemask has an influence on the subjectively perceived walking ability of patients (*question 2*), since it can be assumed that people with a neurological disease notice an impairment of their walking ability by wearing a facemask (*hypothesis 2*). Furthermore, it is known that the subjective perception of the patients might have an influence on gait performance (Hoogkamer et al., 2015; Rosengren et al., 1998), therefore it is relevant to ask about this.

In addition to these two main aspects of the studies, it will also be exploratively investigated whether there is also an influence of wearing a facemask in these clinical tests as well as in walking up and down stairs (*question 3*). It is possible that people with a neurological disorder also show abnormalities in common clinical tests measuring walking ability (stair test, TUG, TUG+DT, 10mWT) (*hypothesis 3*). This finding, albeit exploratory, would provide relevant information for routine clinical testing for all clinicians* who do not have CMill+VR available.

## Test plan

### 2.1. study procedure

The project will investigate the extent to which wearing a facemask affects gait adaptability (CGait) and mobility (clinical parameters) in people with neurological disease.

For this purpose, 50 patients who are undergoing rehabilitation at the neurological rehabilitation center "Godeshöhe" (NRZ) will be asked whether they would like to participate in this study with a measurement point. They will be informed verbally and in written form about the study project. If they wish to participate, they must give their written consent to participation and data processing.

Afterwards, some socio-demographic (age, gender, years of education, height, shoe size, weight, BMI, spectacle wearer, type of visual impairment), and clinical (diagnosis, year of diagnosis, disease severity, fall events in the last 12 months, subjective fear of falling, medication, if applicable, pacemaker settings in people with Parkinson's disease) data are recorded. These are collected in order to be able to describe the sample decisively.

As primary endpoint, the CGait walking test will be completed once with and once without mask by each participant. The order "mask/no mask" will be randomized per participant. Among all participants, the order "mask/no mask" will be equally frequent as the order "no mask/mask". The CGait test is a standard test that is integrated into the diagnostic program of the C-Mill by Motek VR treadmill (DIH GmbH, Cologne, GER). It lasts 20 minutes and includes a series of tasks to test gait adaptability, including crossing obstacles (see Section 2.2.1). In addition, standard motor diagnostic tests (10mWT, TUG, TUG+DT and stair test) are collected. Figure 1 illustrates the planned measurement procedure. Chapter 2.2. gives an overview of the planned data collection


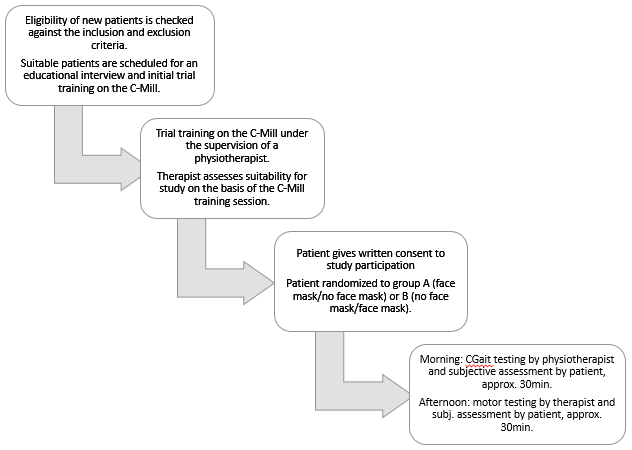


Figure 1: Schematic representation of the study process

### 2.2 Assessments

The data to be collected will be obtained by interview (sociodemographic and clinical data and subjective assessments) and simple clinical examination (motor gait and balance tests). Table 1 summarizes the assessments planned for the study. Most of the data to be collected in the project will be collected in the usual routine. Therefore, the additional burden for the subjects is low (85 min.).

The sociodemographic and clinical data can largely be taken from the digital file. However, they are verified in personal interviews with the patients and the treating physicians (questioning).

The recording of past fall events and fall-associated self-efficacy is carried out by means of standardized questionnaires. The self-evaluation of the test persons is carried out with self-developed questionnaires.

During motor testing, various facets of mobility are recorded using standardized assessments for neurological rehabilitation. The testing is performed by experienced physiotherapists and/or sports scientists who have been familiar with the testing for many years. The assessments cannot be blinded due to staff resources.

Table 1: Planned assessments

| **Tests** | **Form of collection** | **Duration** | **Routine** | **T0** |
| --- | --- | --- | --- | --- |
| Sociodemographic data (total duration approx. 7 min.) | | | | |
| Age in years | File/ Questioning | < 1min. | x | x |
| Gender | File/ Questioning | < 1min. | x | x |
| Training years | File/ Questioning | < 1min. | x | x |
| Weight in Kg | File/ Questioning | < 1min. | x | x |
| Body height in cm | File/ Questioning | < 1min. | x | x |
| Body Measure Index, BMI | Is calculated from height and weight (formula of WHO )^[[1]](#footnote-1)^ |  |  |  |
| Shoe size | Survey | < 1min. |  | x |
| Spectacle wearer | Survey | < 1min. |  | x |
| Type of visual impairment | Survey | < 1min. |  | x |
| Clinical data (total duration approx. 13 min.) | | | | |
| Diagnosis incl. progression form/ type | File/ Questioning | < 1min. | x | x |
| Time since diagnosis (in months) | File/ Questioning | < 1min. | x | x |
| Severity of illness (Expanded Disability Status Scale, EDSS Kurtzke, *Neurol,* 1983; Hoehn & Yahr Scale.  Hoehn & Yahr, Neurol, 1967) | File/survey for motor. Testing | 5 min. | x | x |
| Fall events in the last 12 months | Survey | < 1min. |  | x |
| Fall-associated self-efficacy (Activities-Specific Balance Confidence Scale, ABC *Schott, Z Gerontol Geriat, 2008*). | Interviews | 5 min | x | x |
| Motor data (total duration 45 min., 2x each) | | | | |
| Gait adaptability (CGait, Timmermanns et al, *Phys Ther*, 2019). | Motor Test | 20 min | x | x |
| 10m walking distance (10mWT; ) | Motor Test | 5 min. | x | x |
| Functional mobility and balance (Timed Up and Go Test, TUG Mathias et al, *Arch Phys Med Rehabil*, 1986). | Motor Test | 5 min. |  | x |
| Walking with dual task (TUG with dual task, TUG+DT Hofheinz et al., *HeilberufeScience*, 2011). | Motor Test | 10 min |  | x |
| Ascending and descending stairs (acending/ decending stair test Nightingale, *J Rehabil Res Dev*, 2014). | Motor Test | 5 min. |  | x |
| Respondent interview (total duration 20 min.) | | | | |
| Subjective assessment of subjects on performance with/without mask | Survey | 15 min |  | x |
| Subjective assessment of the C-Mill by test persons | Survey | 5 min. |  | x |

*Total duration*: 150 min. of which exclusively study-related testing approx. 85 min.

*The following applies to the assessments*: If individual limitations arise due to the clinical picture or if the patients need a break for any reason, which they do not need to specify, or do not wish to continue the test/questionnaire, the test/questionnaire will be interrupted or terminated.

#### 2.2.1 Primary endpoint

The central parameter of the study is the performance (performance value in %) per CGait subtest in the two difficulty levels of the CGait (if possible for the patients).

The CGait test measures gait adaptability using six different subtests on the CMill + VR treadmill. A comprehensive description of the CGait is provided in the publication by Timmermans et al. (2019).

During the test, the subjects are safeguarded by a harness and the comfortable walking speed is determined at the beginning of the test. This speed is maintained throughout all tasks. In the planned study project, it will also be maintained for the test with and without mask. All tasks of the CGait will be completed by the subjects first with an easy and then with a hard level of difficulty. The subjects are instructed both via an integrated screen and by the therapists.

The performance for the six test tasks (Fig. 2) "goal-directed stepping", "tandem walking", "obstacle avoidance", "slalom walking", "speed adaptations", is measured. It is determined by the proportion of steps where the center of pressure was within the projected area during the mid-stance phase (± half of the foot size for goal-directed stepping and obstacle avoidance). In the "walking with suddenly shifting obstacles and targets" task, the weighted average of correct steps and correctly avoided obstacles is calculated. For each of the six tasks a performance value between 0 and 100% is obtained.

Figure 2: Six subtests of the CGait test from Timmermans et al., 2019.

The performance values of each subtest are compared per condition (1: with facemask, 2: without facemask) and for each difficulty level (easy, hard).

#### 2.2.2 Secondary endpoints

However, since clinically observed improvement is not only a matter of standardized, objective measurements, but also of patients' self-assessment, which may also have an influence on performance, patients' subjective assessment of whether wearing a facemask has an influence on subjectively perceived walking ability is recorded by means of a survey and evaluated descriptively.

As further secondary endpoints, the central tendencies of the execution times per condition ("wearing a facemask" and "not wearing a facemask") for the respective clinical parameters (10mWT, TUG, TUG+DT and stair test) will be compared.

### 2.3 Blinding

There is no blinding of test and/or training instructors.

### 2.4 Randomization

The subjects are randomly assigned to the conditions, i.e. the order of testing (condition "with mask/without mask" or "without mask/with mask"). Both conditions should occur equally often among all participants.

### 2.5 Statistical evaluation

The collected, pseudonymized data is recorded digitally (dual control principle).

Data analysis is performed using common analysis software IBM SPSS Statistics 27.0 (Armonk, NY) and open source software R (R Foundation for Statistical Computing, Vienna).

At the beginning of the analysis, the data are checked with regard to the fulfillment of required prerequisites. The typical statistical procedures are used for the verification.

Frequencies, medians (with minimum and maximum), or means (with standard deviation) are reported to describe the sample, as appropriate.

To answer research *question 1*, whether wearing the facemask (FFP-2 mask) has an effect on the adaptability of gait (measured with the performance scores of the CGait subtests in both difficulty levels) in people with a neurological disorder, a parametric or non-parametric within-group comparison is calculated, as appropriate.

The *2nd study question*, whether wearing a facemask has an influence on the subjectively perceived walking ability of the patients, is presented using descriptive statistics (frequency tables).

In addition to these two central study questions, research *question 3* of the study, whether there is also an influence by wearing a facemask in the clinical tests measuring walking ability (10mWT, TUG, TUG+DT, and stair test), will be explored. For this purpose, parametric or non-parametric within-group comparisons will be calculated for each subtest, as appropriate.

For all within-group comparisons, the significance level is set at *p* ≤ 0.05.

### 2.6. Publications and registration of the study

The study is submitted as a Registered Report to a journal participating in the Center for Open Science (e.g. BMC Medicine). A Registered Report submission means that the protocol (introduction and method) goes through the peer review process at "Stage 1" before data are collected. After an "In Principle Acceptance," researchers are then allowed to begin the study. In "Stage 2" the results and discussion are peer reviewed and as long as the researchers have performed the pre-approved method and protocols, the study is published in the journal. More on Registered Reports can be found here: <https://www.cos.io/initiatives/registered-reports>

The results of the project will also be presented at congresses (WFNR World Congress or German Society for Neurorehabilitation e. V.) as well as in internal trainings.

The study will be registered in the German Register of Clinical Studies (DRKS). The finalization of the registration will take place after a positive vote by the Ethics Committee (not possible otherwise at the DRKS).

## Total planned duration

The following diagram (Fig. 1) represents the planned test plan of the study project.

Figure 3 Test plan for the study project

## Subject selection

### 4.1 Subject recruitment

Neurological patients are recruited during their inpatient rehabilitation stay at the NRZ "Godeshöhe".

### 4.2 Inclusion and exclusion criteria

Patients will be included or must be excluded from the study according to the following criteria:

| Inclusion criteria | Exclusion criteria |
| --- | --- |
| - A neurological diagnosis (Parkinson's disease or multiple sclerosis) confirmed by the attending physician. - the test persons are capable of being informed - the subjects give their written consent to participate in the study | - no existing contraindications against testing on the C-Mill+VR treadmill - Other neurological or psychiatric diseases that influence the ability to be informed, the test comprehension as well as the training security or have an additional influence on the motor function - insufficient German language skills to follow instructions and tests - Visual or auditory impairment (cannot follow tasks) - >135kg body weight and >2m body height (device restriction) - Open wounds in the area of the C-Mill safety belt |

### 4.3 Sample size

The planned project is to be implemented with the support of 50 test persons. This is considered a feasible sample size for the intended study period. No a priori case number calculation was performed, since no studies are available so far on the basis of which necessary à priori assumptions can be made. Due to the novelty of the topic, clinically relevant minimum differences for the intended outcome parameters have not yet been established. The planned study will make a relevant contribution to this point.

Nonetheless, a sensitivity power analysis showed that with this feasible sample size, the analysis will have a power of 0.8, will have an effect size of *d* = 0.4, and will have a power of 0.95 to identify effect sizes of *d* = 0.52 (see Fig.4)


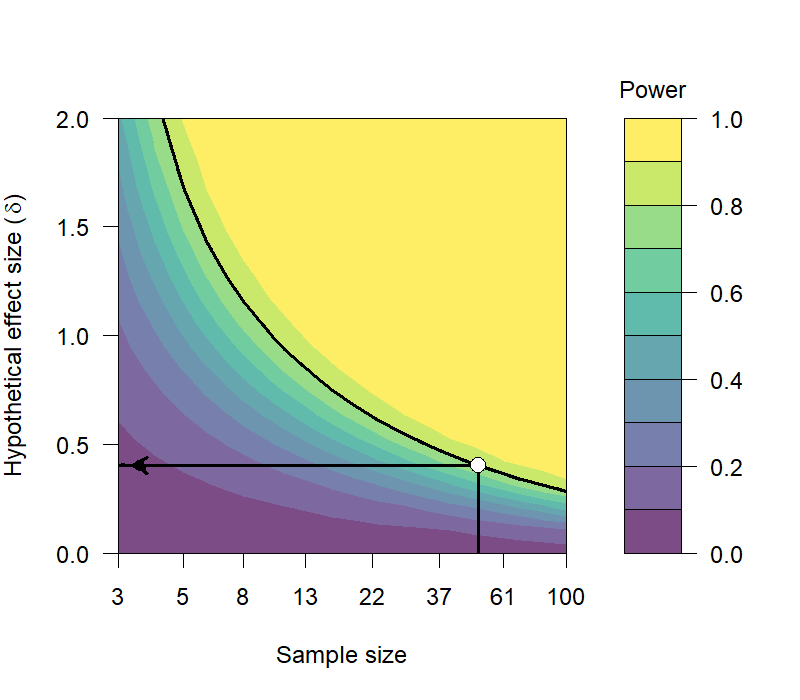

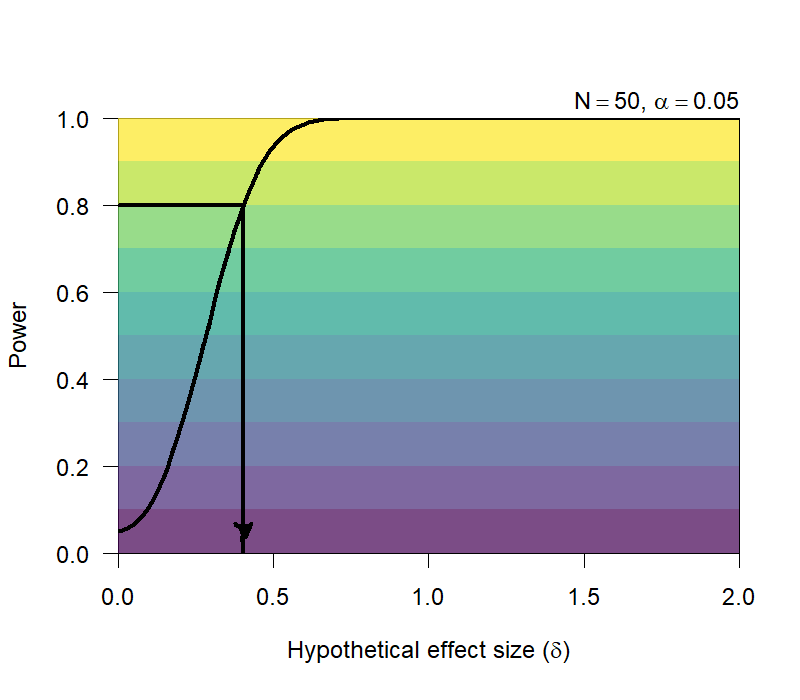
Figure 4: Results of the sensitivity power analysis.

### 4.4 Information and informed consent

Suitable patients are contacted, informed and written consent is obtained. The corresponding procedure is described in detail below.

#### 4.4.1. Contacting

Upon receipt/admission of neurological patients, potential eligibility is determined according to inclusion and exclusion criteria based on the medical record.

Potentially suitable subjects will be informed about the study project in a personal meeting between the subject and the study staff and, if interested, will receive the written information for subjects including the declaration of participation. On the following day, the patients are contacted and asked whether they would like to participate in the study or not.

Patients will be informed verbally and in written form that their participation in the study is voluntary, free of charge and without compensation/reward. They will also be informed that, at the patient's personal request, consent to participate in the study can be withdrawn at any time without giving reasons and without any disadvantage to them.

All patients who wish to participate in the study must be capable of giving consent. The ability of the subjects to give consent is an explicit inclusion criterion of this study. Persons who are not capable of giving consent are excluded from participation.

#### 4.4.2 Reconnaissance

All subjects will be fully informed about the procedure and evaluation in a comprehensible form. The subjects and, if desired by the subjects, their relatives or legal guardians/appointed representatives will be fully informed verbally and in written form about the contents of the study, in particular about the research methods and the interventions as well as their right of withdrawal, and will be informed about the guarantee of data protection. They will also receive detailed participant information.

#### 4.4.3 Consent to study participation

The interested patients will be informed about the motivation, the objectives and the procedure of the planned study in a personal interview between the subject and the study staff as well as in written form. The patients receive the participant information on the study together with the consent form in written form during the informative meeting. Subsequently, the subjects sign an informed voluntary consent form for the study. Subjects will receive a copy of the informed consent form for their personal records.

If an extension to the current project is necessary, it will be submitted to the ethics committee in an amendment for a new decision. If other changes become necessary, the departments affected by the changes (e.g. data protection officers) will be consulted in addition to the ethics committee. If the Ethics Committee recommends that patients also be informed of this amendment, we will do so in consultation with the Ethics Committee (regarding the form).

## Type of testing

It is a human experiment with other testing (neuroscience/ neuropsychology experiment).

## Legal provision and regulations

Concerns CMill with virtual reality (VR):

The C-Mill +VR treadmill by Motek (DIH GmbH, Cologne, GER) is a medical device intended for use in therapy. As intended for the use of medical devices in therapy, the provisions of the Medical Devices Operator Ordinance, or MPBetreibV for short (version dated 27.092016), are observed.

All therapists in the study are instructed in the use of the treadmill by authorized instructors (Peter Wendland, Head of Physiotherapy or Timo Mihm, Deputy Head of Physiotherapy) in accordance with the requirements of §5 Para. 1 MPBetreibV. These are routine procedures for the therapists.

There is also a close exchange with the Motek Company.

The further therapy in both groups corresponds to the established standard of rehabilitation in the NRZ "Godeshöhe". No further study-related intervention in the therapy processes takes place here, so that no additional regulations and prescriptions apply here.

**The entire project** is subject to data protection regulations, which are detailed under point B., 12.

## What preliminary tests have been performed

The CMill + VR is already used in clinical and scientific contexts. In the following, the current state of knowledge, own preliminary work and relevant literature as well as continuing need for research are shown.

### 7.1 Current state of knowledge on the impairment of walking ability by wearing a facemask

It is well known that any masks used to cover the mouth and nose, whether medical or non-medical, cover a significant portion of the face. In particular, FFP2 masks, due to their shape, do protrude into the lower visual field (Kal et al., 2020a, Klatt et al., 2021). It is therefore conceivable that this causes visual field impairment, which has an impact on visual perception and thus gait adaptability (Buckley et al., 2011; Rietdyk & Rhea, 2011), as the lower visual field provides important information for any necessary gait adaptations (e.g., avoiding or stepping over obstacles) and safe walking (Buckley et al., 2011). Limitation in this area due to wearing a facemask could contribute to impaired gait adaptability and thus potentially increased risk of trips and falls, particularly among the elderly and those with previous neurological conditions (Klatt et al., 2021; Callisaya et al., 2020; Rietdyk & Rhea, 2011; Lord et al., 2002). In these people, balance, walking and reaction abilities, among other things, may already be impaired, so that the additional limitations of the visual field due to wearing the facemask and the associated reduced sensory information may no longer be so easily compensated for and the risk of tripping and falling could therefore increase massively (Klatt et al., 2021; Kal et al., 2020a; Yakubovich et al., 2020; Callisaya et al., 2020).

An initial scientific debate has arisen about what affected people might be advised to do to compensate for a perceived increased risk of falling. However, it is clear from the theory-based discussion that clear and personalized recommendations are currently difficult. This also lacks actual practical research that would reveal prevalences and impaired functions in affected individuals and from which initial recommendations could be derived. Educational approaches could also be relevant, but for such educational approaches it is relevant to know who actually belongs to the affected persons and which aspects of gait adaptability are affected. For this purpose, it is relevant not only to discuss this question theoretically, but to collect actual prevalences, for example in the neurological population.

### 7.2 Own investigations and relevant literature

Own work of the cooperation partner: McCrum, 2020

References: Klatt et al, 2021; Kal et al, 2020a; 2020b; Yakubovich et al, 2020; Callisaya et al, 2020.

### 7.3 Continuing need for research

1. To clarify whether or not wearing a facemask actually makes a difference in gait adaptability in the cohort of neurologically predisposed individuals.
2. To understand how affected individuals subjectively assess their walking ability with and without facemask.
3. Which aspects of gait adaptability are most likely to be affected by wearing a facemask.
4. To investigate whether wearing a facemask may also have an effect on the results of common, clinical mobility assessments.
5. To derive recommendations for affected persons and clinicians.

## Pharmacological-toxicological testing

Not applicable

## Possible complications or risks

No increased risk is expected from participation in the study during the planned examinations. Also, no complications in the execution and follow-up problems for the test persons and relatives are expected.

However, the neurological patient population to be worked with in this study may be limited in physical and mental capacity, which could lead to various disease-related risks, e.g., loss of balance with falls, difficulties in orientation, circulatory problems, mood swings, medication fluctuations, etc. However, there is no increased risk here due to study participation. The patients are supervised by experienced neurorehabilitation therapists who are familiar with the clinical picture, can assess and avert possible risks and supervise the use of the training equipment.

Furthermore, the C-Mill by Motek has an extra belt safety system for patients, as well as railings on both sides, an emergency stop switch and a light barrier that prevents patients from getting too far towards the end of the treadmill. These devices are for the safety of the patients. All patients are supervised in individual therapies, so that they are in direct contact with experienced therapists.

## Risk-benefit analysis

The measures take place within the framework of inpatient, neurological rehabilitation. Side effects, which lie exclusively in the applied test procedures, are not to be expected. The patients are continuously guided and observed by experienced therapists/testers. Excessive stress is not to be expected and would be prevented by the respective therapists/testers.

If it could be shown by this study that wearing a facemask has an influence on the gait safety of people with a neurological disease, this would be an important finding for the affected persons as well as for those who work with the affected persons in the care context in order to offer them assistance and to adapt the care and/or therapy accordingly (e.g. to perform gait training with facemask).

In addition, these findings could be used as a basis for developing possible compensation strategies. Even if the CoVID-19 pandemic should end and the wearing of a facemask is no longer necessary, there are still situations in the clinical environment where neurological patients may have to wear a facemask. Further infectious events cannot be ruled out in the future and these findings could be useful for this as well. However, as long as the CoVID-19 pandemic continues, fall prevention, which includes risk assessment for specific groups, is highly relevant to relieve the burden on the healthcare system.

## Interim evaluation and termination criteria

No interim evaluation is planned.

In case of unexpected undesirable side effects, which, however, are not expected to occur, the study will be terminated.

At the personal request of the patient, the discontinuation can be carried out at any time without giving reasons and without any disadvantage for the patient.

## Data sources, data management and data protection

### 12.1. Data source

No existing data are evaluated, but motor, sociodemographic and health-related/clinical data are newly collected. For detailed information on the individual data, please refer to "2.2. Assessments".

### 12.2 Data holding entity

NRZ "Godeshöhe" GmbH

Waldstraße 2-10 ***∙*** 53177 Bonn

All included patients will be recorded in an electronic case report form (eCRF), which will be maintained by the researchers involved in the study. All data concerning the patients will be either digitally recorded directly or transferred from paper versions (copies of motor (routine) tests and questionnaires). The study data will be collected using the established data management platform Castor (https://www.castoredc.com/). This data management platform complies with information security guidelines (ISO 27001; https://www.castoredc.com/security-statement/), which are regularly updated to meet the latest requirements. The data servers comply with applicable guidelines (ISO27001, ISO9001) and national or international standards (HIPPA, NEN7510). During data dormancy, the data stored there are encrypted. During data processing, the data is decrypted, whereby different decoding levels can be defined. Each user receives an individual, password-protected user account through which the data is managed. The respective study director determines who has access to the data. The collected data remain the property of the data holding institutions (see above).

The following persons are responsible for data processing:

Mareike Eschweiler, MSc.

Head of Therapeutic Sciences

NRZ "Godeshöhe" GmbH

Waldstraße 2-10

53177 Bonn

Phone +49 228 381 - 559

Email: [mareike.eschweiler@go.johanniter-kliniken.de](mailto:mareike.eschweiler@go.johanniter-kliniken.de)

Dipl. Psych. Jochen Saliger

Management cognitive rehabilitation

NRZ "Godeshöhe" GmbH

Waldstraße 2-10

53177 Bonn

Phone +49 228 381 - 702

E-mail: jochen.saliger@go.johanniter-kliniken.de

The following data protection officer is also responsible for data protection issues and can be contacted by volunteers:

NRZ "Godeshöhe" gmbH

Data Protection Officer

Waldstraße 2-10

53177 Bonn

Phone: +49 228 381-643

E-mail: datenschutz@go.johanniter-kliniken.de

### 12.3 Data protection

#### 12.3.1 Positive opinion of the data protection officers

The data protection officer of NRZ "Godeshöhe" e.V. has received the ethics application and the declaration of participation for study participants, has checked it with regard to data protection law and approved it after correction, if necessary.

In addition, compliance with the German Data Protection Regulation (DSGVO), the German Federal Data Protection Act (BDSG), the North Rhine-Westphalia Health Data Protection Act and the revised Declaration of Helsinki in the current version of Fortaleza (Brazil), 2013 is fully ensured.

#### 12.3.2 Evaluation with (de facto) anonymized data

Data collection in Castor is pseudonymized by means of the study ID (see section 11.3.3). The protocol sheets of the tests as well as copies of the protocol sheets of the motor (routine) tests and the questionnaires are immediately pseudonymized so that further data processing is de facto anonymized.

There is a key list in the NRZ for the participants included there. The list provides information about the affiliation of the subject IDs to the individual study participants. This list is kept by Ms. Eschweiler. The key list is kept in a lockable cabinet; however, it is kept separately from the protocol sheets. The storage of such a key list is justified by the obligation to provide information to the test persons. After completion of the data collection, this key list will be destroyed immediately.

#### 12.3.3 Preventing the identification of persons

All subjects participating in the study receive an ID consisting of letters and numbers. The letters are the same for each subject and refer to a study code, CMill-NMS for C-Mill by Motek with/without facemask. The number occurs only once and refers to the number of patients. Consequently, it can range from 001 to 50. The numbering of the patients is in ascending order.

#### 11.3.4 Accessibility of the data for third parties

Access to the pseudonymized study data is restricted to employees of the NRZ "Godeshöhe" who are directly involved in the study and are sworn to secrecy, as well as to Dr. Giannouli and PD Dr. McCrum. However, it is planned to make the anonymized data accessible to other scientists in the sense of "open data availability" after completion of the study. This corresponds to the requirements of the planned preregistration and is nowadays a quality criterion of comprehensible scientific practice. The anonymized data will be made available in a portal especially for public scientific datasets, e.g. Science Framework or Zenodo. Here we are also happy to follow alternative recommendations by the ethics committee.

Furthermore, the raw data will be handled as follows: The socio-demographic and clinical data will be recorded directly in the data management system Castor, anonymized. The protocol sheets of the tests and the questionnaires of all study participants are stored pseudonymously in lockable cabinets in the office of Ms. Eschweiler for the time of data collection and archived in the NRZ "Godeshöhe" according to the requirements of good scientific practice (GPS). The documents will be destroyed after ten years in accordance with the GPS. The material will not be passed on to third parties.

All study data will be successively recorded, in parallel with ongoing recruitment, in the Castor data management platform (see section 11.2), which, as described above, complies with the relevant data security regulations. Furthermore, no documents containing personal or health-related information are stored in online data backup systems (e.g. Dropbox, Google Drive, iCloud).

The subsequent publication of the results in international journals or the presentation at congresses will be completely anonymous. A personal reference will not be possible.

All persons involved in the study are bound to secrecy. Compliance with the German Data Protection Regulation (DSGVO), the German Federal Data Protection Act (BDSG), the North Rhine-Westphalia Health Data Protection Act and the revised Declaration of Helsinki in the current version of Fortaleza (Brazil), 2013 is fully ensured.

Patients will receive detailed information about the processing of their data and the persons responsible for data processing, as well as contact information for the NRZ data protection officer and the data protection supervisory authority of the state of North Rhine-Westphalia. In addition, it is emphasized that patients have the right to inspect their data collected during the study at any time and to request deletion if necessary.

## Subject education and consent

All study participants will be fully and comprehensibly informed about the procedure and evaluation. The study participants and, if desired by the study participants, also their relatives will be fully informed verbally and in written form about the contents of the study, in particular about the study methods and their right of withdrawal, and will be informed about the guarantee of data protection. They will also receive detailed participant information (see Appendix I).

Interested patients will be informed in a personal meeting between the study participant and the study management as well as in written form about the motivation, the objectives and the procedure of the planned study project. The patients will receive the participant information on the study together with the informed consent form (see Appendix I) in written form. Study participants will then sign an informed voluntary consent form for the study. Study participants will receive a copy of the informed consent form for their personal records. The informed, written consent forms the legal basis for the intended data collection and processing within the scope of the study (cf. Article 6 No. 1 DSGVO).

Should an extension to the project now applied for become necessary, these will be submitted to the ethics committee in an amendment for a renewed decision. Should other changes become necessary, the departments affected by the changes (e.g. data protection officers) will be consulted in addition to the ethics committee. If the Ethics Committee recommends that patients also be informed of this amendment, we will do so in consultation with the Ethics Committee (regarding the form).

## Insurance coverage

## No additional insurance is taken out for probationers. However, in the event of an injury caused by negligence, patients could make a claim for compensation, which would be covered by the liability insurance of the rehabilitation center.

# C**. PATIENT INFORMATION AND** CONSENT **FORM**

see Annex I

# D. SIGNATURES AND DATE

We hereby declare our consent to the implementation of the study: "Influence of wearing an oral-nasal protection on the gait adaptability of people with a neurological disease" in the NRZ "Godeshöhe", Bonn- Bad Godesberg.

Mareike Eschweiler, MSc.

Head of Therapeutic Sciences

Prof. Dr. med. Hans Karbe

Medical Director of the Neurological Rehabilitation Center Godeshöhe GmbH

Klaus Köhring

Clinic Director of the Neurological Rehabilitation Center Godeshöhe GmbH

**Date:** Bonn- Bad Godesberg, the 22.06.2021

# Literature

Beghi, E., Gervasoni, E., Pupillo, E., Bianchi, E., Montesano, A., Aprile, I., Agostini, M., Rovaris, M., Cattaneo, D., & NEUROFALL Group (2018). Prediction of Falls in Subjects Suffering From Parkinson Disease, Multiple Sclerosis, and Stroke. Archives of physical medicine and rehabilitation, 99(4), 641-651. https://doi.org/10.1016/j.apmr.2017.10.009

Buckley, J. G., Timmis, M. A., Scally, A. J., & Elliott, D. B. (2011). When is visual information used to control locomotion when descending a kerb?. PloS one, 6(4), e19079. https://doi.org/10.1371/journal.pone.0019079

Bundesregierung (2021d). Video conference of the Federal Chancellor with the heads of government of the Länder on March 22, 2021-DECISION. Available at: https://www.bundesregierung.de/resource/blob/997532/1879672/2854753dbc7549432db7f0bba94e8c0f/2021-03-22-mpk-data.pdf?download=1, last access 23.03.2021

Bundesregierung (2021c). How masks protect us in everyday life. Available at: https://www.bundesregierung.de/breg-de/aktuelles/regelung-zu-masken-1842704, last access 19.03.2021.

Bundesregierung. (2021b). Video conference of the Federal Chancellor with the heads of government of the Länder on February 10, 2021-Decision. Available at: https://www.bundesregierung.de/resource/blob/975226/1852514/508d851535b4a599c27cf320d8ab69e0/2021-02-10-mpk-data.pdf?download=1, last access: 19.03.2021

Bundesregierung. (2021a). Video conference of the Federal Chancellor with the heads of government of the federal states on January 19, 2021-Decision. Available at: https://www.bundesregierung.de/resource/blob/975226/1840868/1c68fcd2008b53cf12691162bf20626f/2021-01-19-mpk-data.pdf?download=1, last access: 19.03.2021

Bundesregierung. (2020j). Video conference of the Federal Chancellor with the heads of government of the Länder on November 25, 2020-Decision. Available at: https://www.bundesregierung.de/resource/blob/975226/1820174/fd9794fa8b8e0ec555f005677509c242/2020-11-25-mpk-beschluss-data.pdf?download=1, last access: 19.03.2021

Bundesregierung. (2020i). Video conference of the Federal Chancellor with the heads of government of the federal states on October 28, 2020-Decision. Available at: https://www.bundesregierung.de/resource/blob/975226/1805024/5353edede6c0125ebe5b5166504dfd79/2020-10-28-mpk-beschluss-corona-data.pdf?download=1, last access: 19.03.2021

Bundesregierung. (2020h). Telephone Switching Conference of the Chief of the Chancellor's Office with the Heads of the State and Senate Chancelleries of the Länder on October 7, 2020-Decision. Available at: https://www.bundesregierung.de/resource/blob/975228/1796766/65f8f7a714ddb688953c4c0c6790dcd8/2020-10-07-beschluss-cds-corona-data.pdf?download=1, last access: 19.03.2021

Bundesregierung. (2020g). Chancellor's video conference with heads of state governments on September 29, 2020-Decision. Avaliable at: https://www.bundesregierung.de/resource/blob/975226/1792238/bbe262252712bf09bbb85f93effa9b15/2020-08-29-beschluss-mpk-data.pdf?download=1, last access: 19.03.2021

Bundesregierung. (2020f). Telephone Switching Conference of the Federal Chancellor with the Heads of Government of the Länder on August 27, 2020-Decision. Available at: https://www.bundesregierung.de/resource/blob/975226/1780568/2f9c77a8e8a549bcac8123fbeff4ee27/2020-08-27-beschluss-mpk-data.pdf?download=1, last access: 19.03.2021

Bundesregierung (2020e). The "AHA rules" in the new daily routine. Available at: https://www.bundesregierung.de/breg-de/themen/coronavirus/die-aha-regeln-im-neuen-alltag-1758514, last access 19.03.2021

Bundesregierung. (2020d). Telephone Switching Conference of the Federal Chancellor with the Heads of Government of the Länder on April 15, 2020-Decision. Available at: https://www.bundesregierung.de/breg-de/themen/coronavirus/bund-laender-beschluss-1744224, last access: 19.03.2021

Bundesregierung. (2020c). Meeting of the Chancellor with the Heads of Government of the Länder of 22.03.2020-Decision. Available at: https://www.bundesregierung.de/breg-de/themen/coronavirus/besprechung-der-bundeskanzlerin-mit-den-regierungschefinnen-und-regierungschefs-der-laender-vom-22-03-2020-1733248, last access: 19.03.2021

Bundesregierung (2020b). Guidelines for Combating the Corona Epidemic, March 16, 2020. available at: https://www.bundesregierung.de/breg-de/themen/coronavirus/leitlinien-zum-kampf-gegen-die-corona-epidemie-vom-16-03-2020-1730942, last access, 03/19/2021.

Bundesregierung (2020a). Meeting of the Chancellor with the Heads of Government of the Länder on March 12, 2020-Decision. Available at: https://www.bundesregierung.de/breg-de/themen/coronavirus/beschluss-zu-corona-1730292, last access 19.03.2021

Callisaya, M., Hill, K., Hill, A.M., Mackintosh, S., Batchelor, F., Said, C.M., Sherrington, C., Dawson, R., West, C., & Fu, S. (2020). Rapid Response: Facemasks and risk of falls - a vision for personalised advice and timing? Available from: https://www.bmj.com/content/371/bmj.m4133/rr, last access 25.02.2021

Dias, N., Kempen, G. I., Todd, C. J., Beyer, N., Freiberger, E., Piot-Ziegler, C., Yardley, L., & Hauer, K. (2006). Die Deutsche Version der Falls Efficacy Scale-International Version (FES-I) [The German version of the Falls Efficacy Scale-International Version (FES-I)]. Zeitschrift fur Gerontologie und Geriatrie, 39(4), 297-300. https://doi.org/10.1007/s00391-006-0400-8

Freund, A. (2021). Is the end of the coronavirus pandemic in sight? Available at: https://www.dw.com/en/is-the-end-of-the-coronavirus-pandemic-in-sight/a-56740737, last access 19.03.2021.

Guralnik, J. M., Ferrucci, L., Pieper, C. F., Leveille, S. G., Markides, K. S., Ostir, G. V., Studenski, S., Berkman, L. F., & Wallace, R. B. (2000). Lower extremity function and subsequent disability: consistency across studies, predictive models, and value of gait speed alone compared with the short physical performance battery. The journals of gerontology. Series A, Biological sciences and medical sciences, 55(4), M221-M231. <https://doi.org/10.1093/gerona/55.4.m221>

Hoehn, M.M., Yahr, M.D. (1967) Parkinsonism: onset, progression and mortality. In: Neurology. 17(5), 427- 442

Hofheinz, M., Schusterschitz, C., & Mehrholz, J. (2011). The Timed Up and Go test with additional motor and cognitive task - a suitable test to assess the risk of falls? HeilberufeSCIENCE, 2 (1): 31-37. DOI 10.1007/s16024-011-0108-6.

Hoogkamer, W., Bruijn, S. M., Potocanac, Z., Van Calenbergh, F., Swinnen, S. P., & Duysens, J. (2015). Gait asymmetry during early split-belt walking is related to perception of belt speed difference. Journal of neurophysiology, 114(3), 1705-1712. https://doi.org/10.1152/jn.00937.2014

Hunter, S. W., Divine, A., Frengopoulos, C., & Montero Odasso, M. (2018). A framework for secondary cognitive and motor tasks in dual-task gait testing in people with mild cognitive impairment. BMC geriatrics, 18(1), 202. https://doi.org/10.1186/s12877-018-0894-0

Kal, E. C., Young, W. R., & Ellmers, T. J. (2020a). Facemasks, vision, and risk of falls. BMJ (Clinical research ed.), 371, m4133. https://doi.org/10.1136/bmj.m4133

Kal, E. C., Young, W. R., & Ellmers, T. J. (2020b). Rapid Response: Authors' response to: "Facemasks and risk of falls - a vision for personalised advice and timing?" by Callisaya et al. Available from: https://www.bmj.com/content/371/bmj.m4133/rr-1, last access 25.02.2021.

Klatt, B. N., & Anson, E. R. (2021). Navigating Through a COVID-19 World: Avoiding Obstacles. Journal of neurologic physical therapy : JNPT, 45(1), 36-40. https://doi.org/10.1097/NPT.0000000000000338

Kurtzke J. F. (1983). Rating neurologic impairment in multiple sclerosis: an expanded disability status scale (EDSS). Neurology, 33(11), 1444-1452. https://doi.org/10.1212/wnl.33.11.1444

State of NRW (2021). Ordinance on protection against new infections with the coronavirus SARS-CoV-2 (Corona Protection Ordinance - CoronaSchVO). Available at: https://www.land.nrw/sites/default/files/asset/document/2021-03-05_coronaschvo_ab_08.03.2021_lesefassung.pdf

Lord, S. R., Dayhew, J., & Howland, A. (2002). Multifocal glasses impair edge-contrast sensitivity and depth perception and increase the risk of falls in older people. Journal of the American Geriatrics Society, 50(11), 1760-1766. https://doi.org/10.1046/j.1532-5415.2002.50502.x

Mathias, S., Nayak, U. S., & Isaacs, B. (1986). Balance in elderly patients: the "get-up and go" test. Archives of physical medicine and rehabilitation, 67(6), 387-389.

McCrum, C. (2020). Rapid Response: Walking slower increases anterior stability to a trip: a consideration for facemasks and falls risk. Available from: https://www.bmj.com/content/371/bmj.m4133/rr-0, last access 25.02.2021.

Nightingale, E. J., Pourkazemi, F., & Hiller, C. E. (2014). Systematic review of timed stair tests. Journal of rehabilitation research and development, 51(3), 335-350. https://doi.org/10.1682/JRRD.2013.06.0148

Rapp, K., Freiberger, E., Todd, C., Klenk, J., Becker, C., Denkinger, M., Scheidt-Nave, C., & Fuchs, J. (2014). Fall incidence in Germany: results of two population-based studies, and comparison of retrospective and prospective falls data collection methods. BMC geriatrics, 14, 105. https://doi.org/10.1186/1471-2318-14-105

Rietdyk, S., & Rhea, C. K. (2011). The effect of the visual characteristics of obstacles on risk of tripping and gait parameters during locomotion. Ophthalmic & physiological optics : the journal of the British College of Ophthalmic Opticians (Optometrists), 31(3), 302-310. https://doi.org/10.1111/j.1475-1313.2011.00837.x

Robert Koch Institute (RKI). (2021): Coid-19: case numbers and Germany and worldwide. Available at: https://www.rki.de/DE/Content/InfAZ/N/Neuartiges_Coronavirus/Fallzahlen.html, last access 19.03.2021.

Rosengren, K. S., McAuley, E., & Mihalko, S. L. (1998). Gait adjustments in older adults: activity and efficacy influences. Psychology and aging, 13(3), 375-386. https://doi.org/10.1037//0882-7974.13.3.375

Timmermans, C., Roerdink, M., Janssen, T.W.J., Beek, P.J., & Meskers, CGM. (2019). Automated, standardized, and patient-tailored progressive walking-adaptability training: a proof-of-concept study. Physical Therapy, 99: 882-891.

United Nations, UN (2021). Pandemic will not end for anyone, 'until it ends for everyone'. Available at: https://news.un.org/en/story/2021/01/1082762, last access 19.03.2021

World Health Organization, WHO (2021). Coronavirus disease (COVID-19) Weekly Epidemiological Update and Weekly Operational Update. Available at: https://www.who.int/emergencies/diseases/novel-coronavirus-2019/situation-reports, last access 19.03.2021.

WHO (2020). Mask use in the context of COVID-19. Available at: file:///C:/Users/eschweilerm/Downloads/WHO-2019-nCov-IPC_Masks-2020.5-eng%20(2).pdf, last access: 23.03.2021.

Yakubovich, S., Israeli-Korn, S., Halperin, O., Yahalom, G., Hassin-Baer, S., & Zaidel, A. (2020). Visual self-motion cues are impaired yet overweighted during visual-vestibular integration in Parkinson's disease. Brain communications, 2(1), fcaa035. https://doi.org/10.1093/braincomms/fcaa035

1. https://www.euro.who.int/en/health-topics/disease-prevention/nutrition/a-healthy-lifestyle/body-mass-index-bmi [↑](#footnote-ref-1)
